# IFI35 suppresses the transcription of hepatitis B virus cccDNA minichromosome via promoting HNF4α proteasomal degradation

**DOI:** 10.1186/s12929-026-01239-w

**Published:** 2026-03-30

**Authors:** Nayeon Kim, Jae Jin Shin, Jae Won Oh, Juhee Won, Ah Ram Lee, Mehrangiz Dezhbord, Jeongwoo Park, Ki-Young Lee, Dong-Sik Kim, Kwang Pyo Kim, Kyun-Hwan Kim

**Affiliations:** 1 Department of Precision Medicine, School of Medicine, Sungkyunkwan University, Suwon, Republic of Korea; 2 Department of Applied Chemistry, Institute of Natural Science, Global Center for Pharmaceutical Ingredient Materials, Kyung Hee University, Yongin, Republic of Korea; 3 Department of Biomedical Science and Technology, Kyung Hee Medical Science Research Institute, Kyung Hee University, Seoul, Republic of Korea; 4 Department of Immunology, Samsung Biomedical Research Institute, Sungkyunkwan University School of Medicine, Suwon, Republic of Korea; 5 Division of HBP Surgery and Liver Transplantation, Department of Surgery, Korea University College of Medicine, Seoul, Republic of Korea

**Keywords:** Hepatocyte nuclear factor 4α (HNF4α), Interferon-inducible protein 35 (IFI35), Hepatitis B virus

## Abstract

**Background:**

Hepatitis B virus (HBV) infection is a major health problem with hundreds of millions of people still chronically infected worldwide. Although it is known that cytokines can inhibit HBV replication in infected hepatocytes, much is still unknown about the underlying mechanisms or mediators.

**Methods:**

In this study, we systematically analyzed tumor necrosis factor-alpha (TNF-α) and interferon-gamma (IFN-γ) induced proteins by proteomic analysis and identified interferon-inducible protein 35 (IFI35) as a novel host restriction factor for HBV replication.

**Results:**

Overexpression of IFI35 suppressed HBV transcription, while its reduction showed the opposite effect. Mechanistically, IFI35 regulated the stability of hepatocyte nuclear factor 4α (HNF4α), which is essential for cccDNA transcription. IFI35 did not regulate the transcription of HNF4α but rather promoted its degradation. We found that IFI35 recruits tripartite motif-containing protein 21 (TRIM21), an E3 ubiquitin-protein ligase, for K48-linked ubiquitination of HNF4α. Results were further validated using patient-derived primary human hepatocytes (PHHs) and mouse model of HBV infection. Our results revealed that cytokines, especially IFN-γ, induced IFI35 in hepatocytes. IFI35 promoted the degradation of HNF4α via the TRIM21-mediated ubiquitination, which, in turn, leads to the suppression of cccDNA transcription and viral replication.

**Conclusions:**

Our findings demonstrate that IFI35-TRIM21-HNF4α axis may play a crucial role in TNF-α and IFN-γ induced suppression of HBV. Consequently, these results reveal a novel antiviral action of IFI35 against HBV. These findings may hold value in the development of alternative anti-HBV drugs.

**Supplementary Information:**

The online version contains supplementary material available at 10.1186/s12929-026-01239-w.

## Introduction

Chronic hepatitis B virus (HBV) infection is a significant public health problem, with approximately 257 million people still at risk of developing liver cirrhosis and hepatocellular carcinoma (HCC) [1, 2]. HBV belongs to the hepadnavirus family and has a compact genome structure of 3.2 kb with a partially double-stranded DNA structure, called relaxed circular DNA (rcDNA) [3]. The rcDNA is converted into covalently closed circular DNA (cccDNA), which serves as a complete transcription template by intracellular machinery [4]. Currently, nucleoside analogs and pegylated interferon (IFN) alpha (PEG-IFN-α) have been proven to downregulate HBV infection by suppressing viral replication [5, 6]. However, these therapies are limited by the need for lifelong treatment and potential side effects [7]. Therefore, there is a need to develop efficient therapeutic alternatives to cure HBV infection.

Hepatocyte nuclear factors (HNFs) play a crucial role in regulating the activities of the two enhancers (Enh I and II) and four HBV promoters (precure/core (pre-C/Cp), pre-S1, pre-S2/S, and X promoters), controlling the transcription of cccDNA [8]. Previous studies have shown that HNF4α, HNF1α, and C/EBP bind to the pre-C/C promoter, enhancing the transcription of a genomic 3.5 kb RNA. This RNA serves as a template for reverse transcription, ultimately leading to the formation of rcDNA [9–11]. In liver biopsy specimens collected from chronic HBV-infected patients, a positive correlation between HNF4α expression and HBV replication has been observed [12]. Samples with higher levels of HNF4α expression also contain elevated levels of HBV DNA [12]. These findings suggest that HNF4α may serve as a promising drug target for combating persistent HBV infection.

The human innate immune systems produce cytokines including tumor necrosis factor alpha (TNF-α), interleukins, IFNs, and numerous other mediators [13]. TNF-α and IFN-γ are known to reduce the cccDNA in infected hepatocytes without causing cytolysis [14]. Also, IFN-α upregulates the expression apolipoprotein B mRNA editing cytosine deaminases (APOBECs), which deaminate cccDNA, ultimately leading to its digestion [15]. In host cells, IFN-stimulated genes (ISGs) play a pivotal role in antiviral functions such as clearance of viral infection. Interferon induced protein 35 (IFI35) is one of these ISGs, a 35-kDa protein originally identified as an IFN-γ induced protein by differential screening of the cDNA library of HeLa cells [16]. IFI35 has been associated with the regulation of innate immunity, specifically type I IFNs and proinflammatory cytokines [17–21]. For example, IFI35 plays a critical role in the type I IFN response during foot-and-mouth disease virus infection and has been shown to induce the production of proinflammatory cytokines following highly pathogenic H5N1 virus infection [22, 23]. Previous studies have shown that IFI35 can play either pro- or antiviral roles, depending on the specific viral infection [24–26]. In vesicular stomatitis virus (VSV) infection, IFI35 was reported to support VSV replication by negatively regulating RIG-I activation [24]. However, during bovine foamy virus (BFV) infections, IFI35 efficiently inhibits BFV replication via interaction with the homologous regulatory protein of prototype FV, resulting in the arrest of viral replication and the repression of viral transcription [25, 26]. However, the relationship between IFI35 and HBV is still unknown.

In this study, we identified IFI35 as a protein induced by TNF-α and IFN-γ in human hepatoma cells using proteomic analysis. Overexpression of IFI35 suppressed HBV replication, while its depletion showed the opposite effect. Mechanistically, IFI35 downregulated the stability of HNF4α, which plays an essential role in HBV transcription, in vitro and in vivo. Meanwhile, IFI35 did not regulate the transcription of HNF4α but rather facilitated its proteasome-mediated degradation through K48-linked ubiquitination by recruiting tripartite motif-containing protein 21 (TRIM21), an E3 ubiquitin-protein ligase. These findings unveil a novel mechanism by which IFI35 inhibits HBV replication by recruiting TRIM21 for the degradation of HNF4α. This could be a potential target for the development of anti-HBV drugs.

## Methods

### Cell culture

Human hepatoma cell lines (Huh7, Korean Cell Line Bank, KCLB, 60,104; HepG2, American Type Culture Collection, ATCC, HB8065) were maintained on Dulbecco’s modified Eagle’s medium (DMEM) media (Welgene, gyeongsan, South Korea) supplemented with 10% fetal bovine serum (FBS) and 1% penicillin streptomycin (PS, Gibco, Grand Island, USA) at 37℃ in 5% CO₂ incubator. The human hepatoma HepG2-NTCP cells [27] were maintained in DMEM supplemented with 10% FBS and 2 mM L-glutamine. Transient transfection of cell lines was performed at 70–80% confluency using Lipofectamine 2000 reagent (Invitrogen, Carlsbad, CA, USA) according to the manufacturer’s instructions.

### Cell lysis and protein digestion

Cells were lysed in 100 µL of lysis buffer containing 50 mM TEAB (pH 8.0), 5% SDS, and Halt™ protease inhibitor (1X). Lysis was performed using probe sonication on ice for 2–3 s per cycle. The lysate was centrifuged at 12,000 g for 5 min, and the supernatant was collected for further analysis. Protein concentration was determined using the BCA assay, and 100 µg of protein was prepared for digestion. The protein sample mixed with lysis buffer was sonicated in a bath sonicator for 30 min. Reduction was carried out by adding 5 mM DTT and incubating at 56 °C for 30 min to break disulfide bonds. Alkylation was performed by adding 20 mM iodoacetamide (IAA) and incubating at room temperature in the dark for 30 min. The alkylated proteins were loaded onto S-Trap mini columns (Protifi) following the manufacturer’s protocol. Acidification was achieved by adding phosphoric acid to a final concentration of 1.2% (v/v), followed by mixing with six volumes of S-Trap binding buffer (90% methanol, 100 mM TEAB, pH 7.1). Samples were centrifuged to bind proteins to the column and washed several times with binding buffer. On-column digestion was performed by adding trypsin at a 1:20 enzyme-to-protein ratio in 50 mM TEAB (pH 8.0) and incubating at 37 °C for 12 h. Peptides were sequentially eluted with 50 mM TEAB, 0.2% formic acid, and 50% acetonitrile with 0.2% formic acid. The combined eluates were dried using a vacuum concentrator.

### TMT labeling, fractionation, and desalting

The dried peptides were reconstituted in 100 µL of 100 mM TEAB. For TMT 6plex labeling, peptides from each sample were mixed with TMT reagents reconstituted in anhydrous acetonitrile, following the manufacturer’s protocol. The reaction was carried out at room temperature and quenched with 5% hydroxylamine. TMT-labeled peptides were pooled into a single tube, dried, and subjected to high-pH fractionation.

Pooled peptides were fractionated using high-pH reversed-phase liquid chromatography on an Ultimate 3000 HPLC system (Dionex, Sunnyvale, CA, USA) equipped with a C18 column (4.6 mm × 250 mm, 5 µm particle size). The mobile phase consisted of 10 mM ammonium formate (pH 10) in water as mobile phase A and 10 mM ammonium formate (pH 10) in acetonitrile as mobile phase B. A gradient of 5% to 35% mobile phase B was applied over 110 min at a flow rate of 0.5 mL/min. Fractions were collected every 1 min after 15 min and collected 96 fractions were concatenated into 24 fractions. The fractions were dried and reconstituted in 0.1% formic acid.

Desalting of the fractionated peptides was performed using C18 StageTips (Empore) to remove salts and other impurities. The desalted peptides were dried again and stored at -80 °C until LC–MS/MS analysis.

### Protein identification by LC–MS/MS

Desalted peptides were re-suspended in 0.1% formic acid (FA) and analyzed using an Exploris 480 mass spectrometer (Thermo Fisher Scientific) coupled with a NeoVanquish system (Thermo Fisher Scientific). For proteome profiling, a 3 h linear gradient was applied, starting from 5 to 35% of solvent B, followed by an increase to 80% solvent B, and held for an additional 10 min. The column was re-equilibrated at 1% solvent B before the next injection. The peptides were loaded onto a trap column (2 cm × 75 µm ID) and separated on an EASY-spray analytical column (50 cm × 75 µm ID, 2 µm particle size) at a flow rate of 300 nL/min. Ionization was performed using an EASY-spray source. Data-dependent acquisition (DDA) was used with a 3-s cycle. MS scans were acquired over a mass range of 350 to 1800 Th at a resolution of 120,000 (at m/z 200) with an automated gain control (AGC) target value of 3.0 × 10^6 and a maximum injection time of 50 ms. The most abundant precursors were selected for MS/MS scans with an isolation width of 1.2 Th. MS/MS spectra were acquired at a resolution of 45,000 (at m/z 200) using a normalized collision energy of 32. Dynamic exclusion was set to 30 s to avoid repeated sequencing of the same peptides. The mass spectrometry proteomics data have been deposited to the ProteomeXchange Consortium via the PRIDE partner repository (dataset identifier: PXD066477) [72].

### Raw data processing

Raw files were processed using Proteome Discoverer 3.0 (Thermo Fisher Scientific) with the SEQUEST HT search engine. MS1 mass tolerance was set to 10 ppm, and MS/MS mass tolerance was set to 0.02 Da. TMT 6plex reporter ions were extracted with a mass tolerance of 20 ppm. Search modifications included static carbamidomethylation on cysteine (+ 57.021 Da) and dynamic modifications for methionine oxidation (+ 15.995 Da). TMT 6plex modifications (+ 229.163 Da) on lysine and peptide N-termini were set as fixed modifications. Protein quantification was based on reporter ion intensities from unique and razor peptides. The minimum confidence threshold for peptide identification was set to 1% FDR at both the peptide and protein levels.

### Data analysis

Statistical and bioinformatics analyses were performed using R (version 4.4.1). Pre-processing included median normalization of reporter intensities, followed by imputation of missing values at 50% of the minimum observed value using the "impute" package in R. Statistical significance was assessed using a independent Student’s t-test. Fold changes were calculated using TMT reporter ion intensity.

### Functional enrichment analysis and network analysis

Functional enrichment analysis was conducted using single-sample gene set enrichment analysis (ssGSEA) to identify significant pathways based on the Hallmark database. Only pathways with p-values less than 0.05 were considered. To construct protein–protein interaction networks, proteins associated with significant Hallmark terms were mapped using interaction data from the STRING database. The resulting networks were visualized and refined using Cytoscape (version 3.10.2).

### Isolation of primary human hepatocytes (PHHs) and transfection

PHHs were isolated from patient liver tissues by using a two-step collagenase perfusion method [28]. The liver specimens were perfused through vein vessels on the cut surface of the specimen with cold perfusion buffer supplemented with collagenase (0.5 g/L) and calcium chloride (0.56 g/L). The cells were filtered through stainless steel meshes in two steps (grid size, 250 and 150 μm). The cells were washed twice with cold William's medium. Human liver tissue specimens proved negative for HAV, HBV, HCV, and HDV infection were obtained from therapeutic hepatectomies. Informed consent was obtained from patients before the procedure. PHHs were isolated with the approval from the Institutional Review Boards at Korea University Hospital (IRB No. ED17180). For transient transfection of PHHs, Lipofectamine 3000 reagent and Lipofectamine 2000 (Invitrogen, Carlsbad, CA, USA) were used according to the manufacturer's protocols.

### Plasmids and siRNA transfection

Replication-competent HBV 1.2mer plasmid has been described in a previous paper [29]. For myc-IFI35 plasmid, the IFI35 gene cloned from HepG2 cells was inserted into the pCMV-myc vector (Clone tech, CA, USA). HBV enhancer-luciferase reporters and pHNF4α-Myc were used in our previous reports [30–32]. HBV enhancer I-ΔHNF4α-Luc were cloned into pGL3-basic vector. Plasmids encoding HA-tagged K48, K48R, and K63 ubiquitin mutants were kindly provided by Dr. Jin-Hyun Ahn (Sungkyunkwan University, Korea) [33]. Luciferase reporter plasmids pGL3-PreS1p and pGL3-PreS2p were generously provided by Dr. Kyongmin Kim (Ajou University, Korea) [33]. The siRNAs were used as follows. The negative control was Ambion, siIFI35-1 (#3430–1) was from Bioneer, and siIFI35-2(#S7142) was from Ambion. HBV enhancer II-ΔHNF4α1-Luc and HBV enhancer II-ΔHNF4α1,2-Luc reporter constructs were cloned into the pGL3-Basic vector (Cosmo Genetech). IFI35-Zip-GFP, IFI35-ΔNID2-GFP, and IFI35-ΔNID1-GFP expression plasmids were generated using the EGFP-N1 vector (Cosmo Genetech). Small interfering RNA targeting HNF4α (siHNF4α) was obtained from Bioneer. Lipofectamine RNA/iMAX (Thermo Fisher Scientific, Waltham, MA) was used for siRNA transfection, and Lipofectamine 2000 was used for plasmid transfection. The protocol was carried out according to the company's manual.

### Enzyme-linked immunosorbent assay (ELISA)

Cell supernatants were gathered to determine the level of secreted HBeAg and HBsAg. The supernatant was diluted with PBS and the relative amounts of HBeAg and HBsAg were determined using a kit (Wantai Bio-Pharm, Beijing, China) according to the manufacturer's protocol. GraphPad Prism 10.0.0 (http://www.graphpad.com/) was used to perform statistical analysis.

### Southern and Northern blotting

A detailed method for Southern blotting of core-associated HBV DNA is described in our previous paper [34]. Briefly, cells were harvested and washed with PBS and lysed with HEPES NP-40 lysis buffer. After pelleting, DNase1 was treated at 37 °C for 20 min to remove the transfected plasmids. HBV core particles were isolated by incubation on ice for 1 h using polyethylene glycol (PEG) solution, followed by incubation with proteinase K at 37 °C for 2 h to degrade the capsid structure. Viral DNA was then isolated via phenol–chloroform-isoamyl alcohol (25:24:1, Sigma, USA), followed by precipitation of HBV DNA using ethanol and 3 M sodium acetate. Purified HBV DNA was transferred to Hybond-N + nylon membrane (GE Healthcare, Buckinghamshire, UK) using alkaline transfer method after running on 1% agarose gel. The membranes were then hybridized in Church buffer with HBV specific probes and developed using DIG system (Sigma, USA) according to the manufacturer's protocol. Replication values were obtained by densitometric analysis of grayscale band intensities using MultiGauge software. Experiments were repeated three times independently with similar results. HBV RNA levels were analyzed by Northern blot analysis as follows. Total RNA was extracted using TRIzol reagent (Sigma-Aldrich) according to the manufacturer’s protocol. Total RNA (20 μg) was separated on a 1% formaldehyde agarose gel at 90 V for 3 h and transferred to a nitrocellulose membrane (GE Healthcare) overnight. To detect HBV RNAs, the membranes were hybridized using the same probes as in Southern blotting. 28S and 18S were used as loading controls.

### Western blotting

Cell pellet was lysed with radioimmunoprecipitation assay (RIPA) buffer containing proteinase inhibitor cocktail (Invitrogen) for 20 min on ice. Sample buffer (Bio-Rad) was added to the lysates, boiled at 95 °C for 5 min, and cooled on ice. Samples were run through SDS-PAGE and transferred using Turbo transfer pack (#1,704,156, Bio-Rad, California, USA). Membranes were blocked using 5% skim milk and incubated overnight at 4 °C with a primary antibody solution containing 3% BSA. After washing 3 times with TBS-T, membranes were incubated in a rocker for 1 h with a secondary antibody solution containing 3% skim milk, and developed using ECL. The following antibodies were used: IFI35 (sc-100769, Santa Cruz), HNF4α (H-171, Santa Cruz), HNF1α (Η-140, Santa Cruz), HNF3β (RY-7, Santa Cruz), core protein (B0586, Dako, Carpinteria, CA), TRIM21 (sc-25351, Santa Cruz), β-actin (A5441, Sigma) and GFP (sc-9996, Santa Cruz). Horseradish peroxidase-conjugated (HRP) secondary antibody was purchased from Sigma.

### Immunoprecipitation

The cells were harvested at 48 h post-transfection and lysed with RIPA buffer containing a protease inhibitor cocktail. The lysates were then diluted 1:5 with RIPA buffer and precleaned with protein G-agarose (Roche) for 2 h at 4 °C. The clarified lysates were then incubated with primary antibodies overnight at 4 °C. The immune complexes were precipitated with protein G-agarose for 6 h at 4 °C, washed three times with cold PBS, boiled for 5 min in sample buffer, and analyzed by Western blotting. For immunoblot detection following immunoprecipitation, HRP-conjugated TrueBlot secondary antibodies (Rockland) or VeriBlot secondary antibodies (Abcam) were used to minimize the detection of immunoglobulin heavy and light chains derived from the immunoprecipitating antibodies.

### Ubiquitination assay

A plasmid encoding HA-tagged ubiquitin [35] was co-transfected with the plasmids indicated in the figures. At 43 h post-transfection, MG132 (20 μM) was added for 5 h. The cells were lysed with SDS lysis buffer containing the deubiquitinase inhibitor *N*-ethylmaleimide and boiled for 10 min. The lysates were diluted 1:10 with TBS containing a protease inhibitor cocktail and the deubiquitinase inhibitor *N*-ethylmaleimide, followed by overnight incubation with anti-HA antibody at 4 °C. The levels of polyubiquitination were analyzed by Western blotting with anti-Ubiquitin antibody (sc-8017, Santa Cruz).

### HBV virion production and infection.

HBV virions (genotype D) were isolated and concentrated with 8% PEG 8000 (PEG8000) from the culture medium of HepAD38 cells, and subsequently quantified by HBV-DNA real-time PCR (RT-PCR). The HBV infections were performed as previously described [36]. Briefly, HepG2-NTCP or PHH cells were infected with 1000 genome equivalents per cell (Geq/cell) of HBV in the presence of 4% PEG8000. After 16 h, the infection inoculum was removed, and the cells were washed three times with PBS. Subsequently, the cells were maintained in culture medium containing 2% dimethyl sulfoxide (DMSO, Sigma-Aldrich).

### cccDNA extraction

To isolate protein-free DNA and cccDNA, the Hirt extraction method was employed. Initially, cells were lysed with 1 ml of a solution containing 50 mM Tris–HCl (pH 7.5), 10 mM EDTA, 150 mM NaCl and 1% SDS at room temperature for 30 min. Subsequently, the lysates were treated with 2.5 M KCl, followed by overnight incubation at 4 °C and centrifuged at 13,000 RPM. Only the supernatant was transferred to another tube and subjected to phenol:chloroform extraction twice and chloroform extraction once. After alcohol precipitation, the DNA was dissolved in TE buffer, after which DNA loading dye was added, and the mixture was loaded onto agarose gel. After running the gel, subsequent steps were carried out in the same manner as Southern blotting.

### Real-time PCR

To synthesize cDNA, reverse transcription was performed using High-capacity RNA-to-cDNA kit (# 4,387,406, Thermofisher Scientific,). Real-time PCR was conducted using SYBR green PCR master mix (Applied Biosystems, Foster City, CA). Primers for HNF4α, and HNF3β were described previously [31]. Real-time quantitative PCR amplification was carried out in QuantStudio 3 Real-Time PCR System (Applied Biosystem). The results were calculated by an n-fold difference relative to the calibrator (RQ = 2 − ΔΔCt).

### Luciferase assay

Cells were transfected with plasmids Enh I, Enh II, and myc-IFI35 in HepG2 cells. β-galactosidase plasmid (0.5 μg) was co-transfected as an internal loading control. Luciferase activity was normalized to β-galactosidase activity to account for transfection efficiency. After 48 h of post-transfection, the cells were lysed with lysis buffer. The luciferase activity was determined using the luciferase assay system (Promega) according to the manufacturer's instructions. The luciferase signals were measured in a luminometer. Experiments were repeated three times independently with similar results.

### Generation of TRIM21 knock-out (TRIM21 KO) cell line

TRIM21 KO cell was generated as described in our previous report [37]. Lentivirus was produced after co-transfection with pCMV delta R8.2, pCMV-VSV-G, and pLentiCRISPR V2-TRIM21 (at a ratio of 1:1:1) into the HEK293T cell line using Lipofectamine 2000. At 48 h post-transfection, the supernatant was collected and filtered through a 0.45 µm pore filter. The lentivirus-containing media was then mixed with fresh media supplemented with polybrene (12 µg/ml) in a 1:1 ratio and applied to the HepG2 cell line. Infected cells were subsequently selected at the single-cell level using puromycin (Sigma-Aldrich, Saint Louis, MO, USA) and TRIM21 expression was analyzed by Western blotting.

### RNA stability assay

HepG2 cells were seeded in 6-well plates and transfected with 1 μg of HBV1.2 plasmid and 2 μg of either control vector or IFI35 expression plasmid using Lipofectamine 2000 (Invitrogen) according to the manufacturer’s instructions. At 72 h post-transfection, cells were treated with Actinomycin D (10 μg/mL; Sigma-Aldrich) to inhibit transcription. Total RNA was isolated at 0, 2, 4, and 6 h post-treatment using TRIzol reagent (Thermo Fisher Scientific). HBV RNA levels were analyzed by Northern blot using digoxigenin-labeled probes specific to HBV transcripts. RNA loading was verified by ethidium bromide staining of 28S and 18S rRNA. Band intensities were quantified using MultiGauge software (Fujifilm), and RNA decay kinetics were evaluated by comparing signal intensities over time.

### Hydrodynamic injections in mice

Six-week-old male C57BL/6 mice were purchased from Orient Bio (Suwon, Korea) and maintained at Laboratory Animal Research Center of Sungkyunkwan university. The animal experiment was approved by the Ethical Committee of Sungkyunkwan University Animal Care (Approval number: SKKUIACUC2022-12–23-1). The mice were randomly divided into the empty vector injection group (n = 4) and IFI35 injection group (n = 7). They were hydrodynamically injected with pHBV 1.2mer (20 µg) and empty vector (40 µg) or pMyc-IFI35 (40 µg) via the tail vein. The mice were sacrificed and serum and liver samples were collected at 4 days post-injection.

## Results

### Proteomic profiling revealed IFI35 as a potential anti-HBV protein induced by antiviral cytokines

**Fig. 1:**
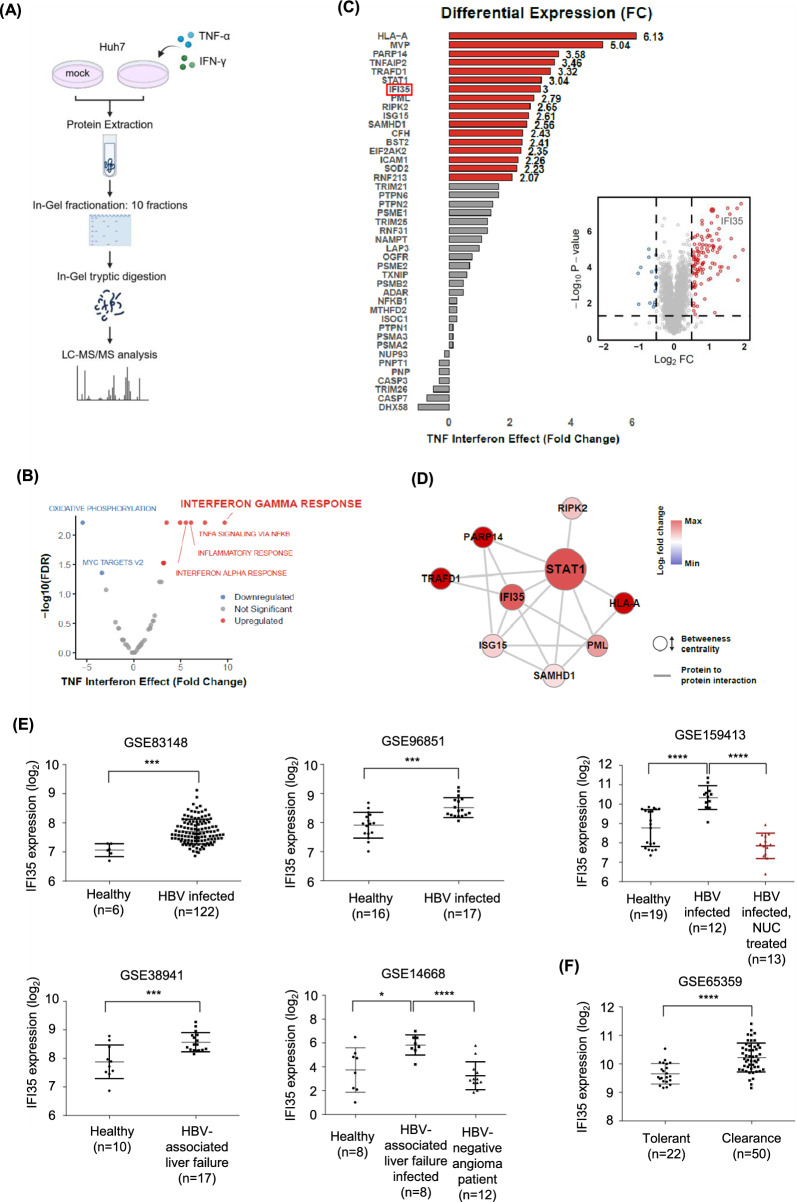
Identification of IFI35 as an interferon-induced protein based on mass spectrometry analysis. **A** Schematic representation of mass spectrometry. **B** Gene Set Enrichment Analysis (GSEA) of significantly enriched pathways in TNF-α and IFN-γ treated Huh7 cells. Hallmark Database was used for GSEA analysis, with FDR ≤ 0.05 and |TNF interferon effect fold change|≥ 3 (**C**) Differential protein expression of Interferon gamma response proteins and volcano plot of differentially expressed proteins in Huh7 cells treated with IFN-γ and TNF-α. The X-axis represents log₂ fold change (FC) and the Y-axis represents –log₁₀ adjusted p-value. Red and blue dots indicate significantly upregulated and downregulated proteins, respectively (cutoffs: |log₂FC|> 0.5, p < 0.05). Gray dots represent genes without significant changes. **D** Protein–protein interaction network of Interferon gamma response proteins. Network was structured based on STRING DB. Protein expression is reflected in the color and Betweenness centrality is applied on size. **E** IFI35 expression in liver tissues from public HBV-related transcriptomic datasets obtained from the Gene Expression Omnibus (GEO). In GSE159413, nucleos(t)ide analogue (NUC)-treated HBV patients are highlighted in red to distinguish them from untreated HBV-infected patients and healthy controls. Expression values represent normalized expression values obtained using GEO2R, and each dot represents an individual sample. **F** IFI35 expression in immune tolerant and clearance phases of HBV infection from dataset GSE65359. Expression values were obtained from GEO2R analysis, and each dot represents an individual sample. Statistical significance was determined by one-way ANOVA with multiple comparisons. ****, P < 0.0001

To identify the novel antiviral proteins responsible for the suppression of HBV by TNF-α and IFN-γ [14], we performed LC–MS/MS analysis on Huh7 cells following treatment with these cytokines, as depicted in Fig. 1A. Using TMT-based quantitative proteomics, we identified a total of 5,598 proteins, which are listed in Supplementary Data 1. To interpret the differences between the two groups, statistical analysis was conducted using a Student’s t-test, followed by single-sample Gene Set Enrichment Analysis (ssGSEA). Untreated Huh7 cells were used as a control. The Hallmark gene set enrichment analysis identified immune-related pathways, including ‘Interferon gamma response,’ ‘TNF-α signaling,’ ‘Inflammatory response,’ and ‘Interferon alpha response,’ as the top enriched processes with significantly high scores. (Fig. 1A). Further analysis through Fig. 1B focused on the pathway with the highest enrichment score, ‘Interferon gamma response,’ to calculate the TNF-IFN effect. This revealed key protein changes in HLA-A, MVP, PARP14, TNFAIP2, TRAFD1, STAT1, and IFI35, highlighting their potential roles in the antiviral response. Therefore, we focused on the ‘Interferon gamma response’ among the upregulated proteins and noted the proteins belonging to this category (Fig. 1C). As depicted in the volcano plot (Fig. 1C), differentially expressed proteins were defined by a log₂ fold change greater than ± 0.5 and an adjusted p-value < 0.05. Among these, IFI35 emerged as one of the most prominently induced proteins, showing a marked increase in expression (log₂ fold change > 1.5, adjusted *p* < 0.001). To analyze the interactions of these proteins, we constructed a protein–protein interaction (PPI) network. The results revealed that IFI35 was associated with most IFN-induced proteins, particularly STAT1, a well-known transcription factor for facilitating IFN-γ-induced immune responses (Fig. 1D) [38]. Based on these preliminary analyses and the lack of previous studies of IFI35 and HBV, we decided to analyze IFI35 as a novel host restriction factor for HBV infection.

First, we analyzed public datasets to determine the expression of IFI35 in HBV-infected patients. By analyzing gene expression profiles using datasets GSE83148, GSE96851 and GSE159413 [39, 40], we found that the expression of IFI35 was significantly higher in HBV-positive patients compared to HBV-negative patients [39, 40]. To investigate the relationship between antiviral therapy and IFI35 expression, we analyzed the GSE159413 dataset, which includes HBV-infected patients with or without nucleoside analogue (NUC) treatment. As shown in Fig. 1E (GSE159413), NUC-treated patients are highlighted in red to distinguish them from untreated HBV-positive individuals. Interestingly, IFI35 expression was significantly reduced in the NUC-treated group, reaching levels comparable to those of uninfected healthy controls. These findings suggest that IFI35 expression is positively associated with active HBV replication and is downregulated upon viral suppression by long-term NUC therapy. We also analyzed the tissue datasets GSE38941 and GSE14668 [41, 42], which were obtained from HBV-negative normal liver donors, liver tissues from HBV-negative angioma patients and patients with HBV-associated liver failure. Again, we found that the expression of IFI35 was significantly increased in patients with HBV-associated liver failure (Fig. 1E). To investigate the correlation between clinical stage of chronic hepatitis B (CHB) patients and IFI35 expression, we analyzed a public dataset of human liver biopsies (GSE:65,359). The results showed that IFI35 was significantly higher expressed in CHB patients with immune clearance phase, where cytokine secretion by immune cells is more active than in immune tolerance phase. These findings suggest that IFI35 expression may be regulated by cytokines and may also play some antiviral role in the immune clearance of HBV (Fig. 1F). Taken together, these results suggest that IFI35 expression may be regulated by viral replication or by antiviral cytokines produced by immune cells and may have antiviral activity.

### IFI35 is primarily induced by IFN-γ, and its overexpression inhibits HBV replication

**Fig. 2:**
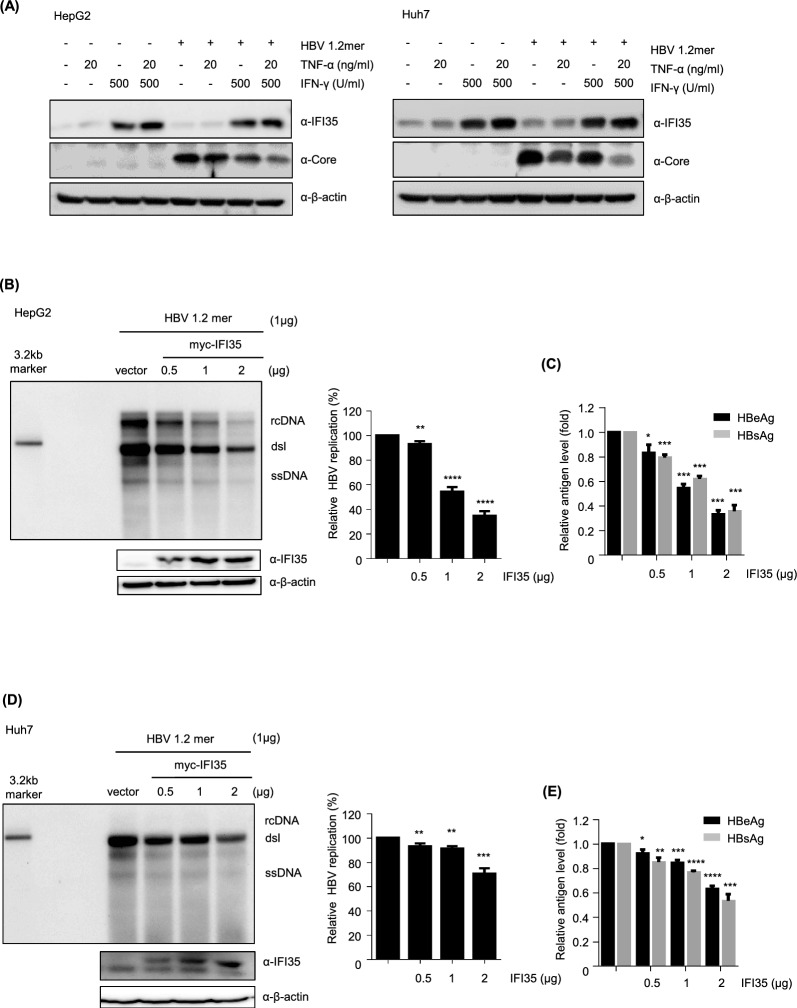
Overexpression of IFI35 suppressed HBV replication. **A** HepG2 (left) and Huh7 (right) cell were transfected either with or without HBV 1.2. After 24 h, TNF-a and IFN-r were treated to confirm the induction of IFI35 and its effect against HBcAg. β-actin was used as a loading control. **B** HepG2 cells were co-transfected with myc-IFI35 plasmids and HBV 1.2. The total amount of transfected DNA per well was equalized by supplementing with empty vector. After 72 h, cells and supernatant were harvested. Cells were lysed to extract core-associated DNA for Southern blot analysis, which assessed the antiviral ability of IFI35. Western blot analysis was performed to confirm the induction of myc-IFI35. β-actin served as the loading control. The intensity of the HBV replication band was quantified and plotted on the right. **C** HBeAg and HBsAg were quantified using ELISA. **D** HBV replication in Huh7 was measured through quantitative analysis of the Southern blot. The intensity of the HBV replication band was quantified and depicted on the right. **E** HBeAg and HBsAg levels in the supernatants were determined by ELISA. The described experiment was independently repeated three times. Data are shown as mean ± standard deviations (SD). The statistical significance of the differences was assessed by the Student t test: *, P < 0.05; **, P < 0.01, ***, P < 0.001

First, to determine which of HBV replication and antiviral cytokines is responsible for IFI35 induction, we examined the expression of IFI35 in HepG2 and Huh7 hepatocytes in the presence or absence of HBV expression and cytokine treatment. We also determined which of the antiviral cytokines, TNF-α or IFN-γ, induced IFI35 more effectively in hepatocytes (Fig. 2A). HBV replication had no effect on IFI35 expression levels in either cell line. Rather, IFI35 expression was predominantly induced by IFN-γ and little by TNF-α. The reduction in core protein is thought to be due to the antiviral action of these cytokines. To investigate whether IFI35 is involved in interferon–mediated antiviral responses against HBV, we examined the induction of IFI35 following interferon treatment. HepG2 cells transfected with HBV 1.2mer were treated with increasing doses of IFN-α or IFN-γ, and IFI35 protein levels were analyzed by immunoblotting. IFN-γ treatment markedly induced IFI35 expression in a dose-dependent manner (Supplementary Fig. 1A). In contrast, IFN-α treatment resulted in only modest upregulation of IFI35 compared with IFN-γ. As a control for interferon signaling activation, the interferon-stimulated gene ISG20 was robustly induced by both IFN-α and IFN-γ treatment. These results indicate that IFI35 is preferentially induced by IFN-γ rather than IFN-α in HepG2 cells, suggesting that IFI35 may play a more prominent role in IFN-γ–associated antiviral responses than in IFN-α–mediated suppression of HBV replication. To determine whether IFN-γ alone suppresses HBV RNA expression, HepG2 cells transfected with HBV 1.2mer were treated with increasing doses of IFN-γ. Northern blot analysis showed that IFN-γ reduced HBV transcription in a dose-dependent manner, with a detectable decrease beginning at 250 U/ml. Consistent with reduced in viral transcription, IFN-γ treatment also decreased the levels of secreted HBeAg and HBsAg levels (Supplementary Fig. 1C). These data indicate that IFN-γ treatment alone is sufficient to suppress HBV mRNA expression in hepatoma cells, and that doses ≥ 250 U/ml can measurably reduce viral transcription (Supplementary Fig. 1B). To investigate the effect of IFI35 on HBV replication, HepG2 and Huh7 cells were co-transfected with replication-competent HBV (HBV1.2mer) and myc-IFI35 plasmids. Southern blot analysis showed that IFI35 significantly inhibited HBV replication in a dose-dependent manner in both HepG2 and Huh7 cells (Fig. 2B and D). Similarly, IFI35 also showed a significant dose-dependent inhibition of HBeAg and HBsAg production in both cell lines (Fig. 2C and E). All these results indicate that IFI35 is mainly induced by IFN-γ and can suppress HBV replication and antigen expression effectively.

### Knock-down of IFI35 promotes viral replication and IFI35 mediates antiviral activity of cytokines

**Fig. 3:**
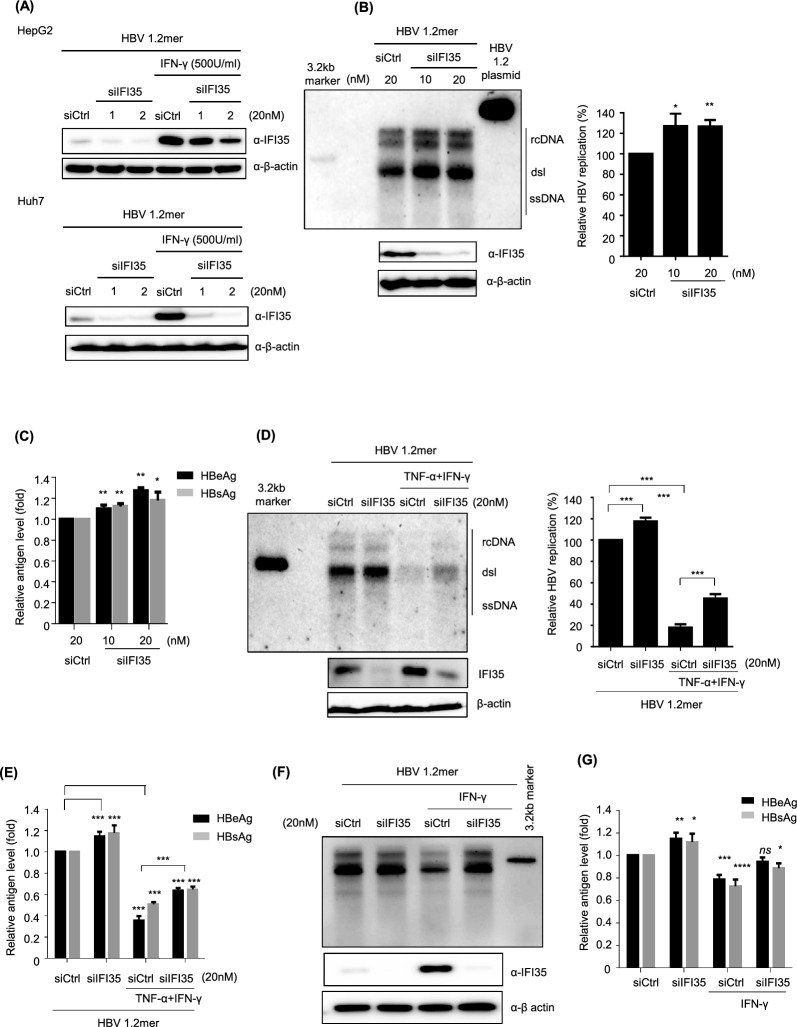
siRNA-mediated downregulation of IFI35 facilitates HBV replication. **A** HepG2 and Huh7 cells were seeded in a 6-well plate. The following day, HepG2 cells were transfected with siRNA. After 8 h, cells were transfected with the 1.2( +) plasmid. After 24 h, IFN-r at 500U/ml was treated, and cells and supernatant were harvested 48 h later. Cells were lysed for western blot analysis to determine the IFI35 amount. β-actin was used as the loading control. **B** Southern and Western blot analyses were conducted after the cotransfection of HBV 1.2 and IFI35 siRNA in HepG2. The amount of replication was graphically represented on the right. A control lane containing 1 ng of HBV1.2 plasmid DNA was included to confirm that the detected HBV DNA was not derived from the transfected plasmid. **C** HBeAg and HBsAg were quantified using ELISA. **D** After cotransfection of HBV 1.2 and IFI35 siRNA, TNF-α and IFN-γ were treated in HepG2. Southern blot and western blot analyses were performed. Viral replication was depicted in the graph on the right. **E** HBeAg and HBsAg levels in the supernatants were determined using ELISA. **F** HepG2 cells were co-transfected with HBV 1.2mer and either control siControl or siIFI35; 20 nM. 6 h post-transfection, cells were treated with IFN-γ (1000 U/ml) three times and harvested at 72 h post-transfection. HBV replication was analyzed by Southern blotting. IFI35 knockdown efficiency was confirmed by immunoblotting. β-actin served as a loading control. **G** Secreted HBeAg and HBsAg levels were quantified and presented as relative levels normalized to the siControl condition. Data are presented as mean ± SD. The statistical significance of the differences was assessed by the Student t test: *, P < 0.05; **, P < 0.01, ***, P < 0.001

The knock-down activity of designed siRNA for IFI35 (siIFI35) was assessed using HepG2 and Huh7 cells. It efficiently down-regulated the expression level of both endogenous and IFN-γ induced IFI35 (Fig. 3A). Knock-down of IFI35 significantly increased the levels of HBV replication (Fig. 3B) and secreted HBsAg and HBeAg antigens (Fig. 3C). To confirm that HBV DNA detected by Southern blot was not derived from transfected plasmid DNA, 1 ng of HBV1.2 plasmid was loaded as a control (Fig. 3B lane 4). Next, to investigate whether the cytokine-induced IFI35 can mediate the antiviral activity of TNF-α and IFN-γ, we treated with TNF-α and IFN-γ in IFI35 knock-down cells. Treatment with TNF-α and IFN-γ strongly suppressed HBV replication; however, viral replication was significantly restored in cells transfected with siIFI35 (Fig. 3D). The protein levels of basal and cytokine-induced IFI35 were markedly reduced by siIFI35 treatment (Fig. 3D). Similarly, the levels of HBsAg and HBeAg also significantly restored by knock-down of IFI35 (Fig. 3E). To determine whether IFI35 contributes to the antiviral activity of IFN-γ, HepG2 cells were co-transfected with HBV 1.2mer and either control siRNA or siIFI35, followed by treatment with IFN-γ (1000 U/ml). IFN-γ treatment markedly reduced HBV replication and decreased the levels of secreted HBeAg and HBsAg in control cells (Fig. 3F and G). Notably, depletion of IFI35 partially restored HBV replication and viral antigen production under IFN-γ treatment, indicating that IFI35 contributes to the antiviral effect of IFN-γ. In contrast, under combined TNFα and IFN-γ treatment, depletion of IFI35 resulted in only limited recovery of HBV replication. These results suggest that the contribution of IFI35 to the antiviral response is more clearly observed under IFN-γ treatment alone than under combined cytokine treatment. Overall, these results indicate that IFI35 is a novel anti-HBV protein and it partly mediates the antiviral activity of TNF-α and IFN-γ.

### IFI35 suppressed HBV in PHH and HepG2-NTCP infection systems

**Fig. 4:**
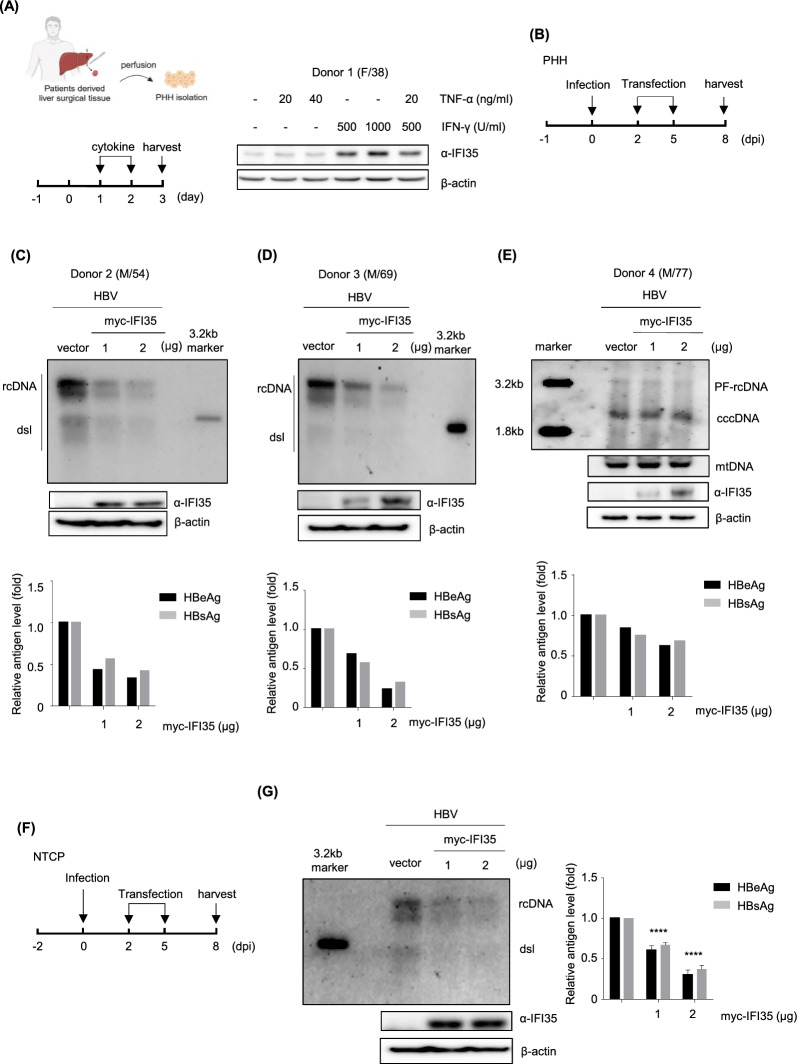
IFI35 repressed HBV replication in infection model. **A**–**D**, **E** Primary human hepatocytes (PHH) were seeded in a collagen-coated 6-well plate. The following day, they were infected with media containing 4% PEG and 2% DMSO mixed with HBV inoculum (1000 Geq). Myc-IFI35 plasmid was transfected on the 2nd and 5th days respectively and harvested on the 8th day. **B** Cells were lysed using the Hirt DNA extraction method, followed by Southern blot analysis. The IFI35 level was confirmed through Western blotting. HBeAg and HBsAg levels in the supernatants were quantified using ELISA. **C**, **D** Encapsidated intermediates were detected by Southern blotting. The levels of HBeAg and HBsAg in culture supernatants were determined by ELISA. **F** HepG2-NTCP cells were infected with HBV virions (1000 Geq) with media containing 4% PEG and 2.5% DMSO. Myc-IFI35 plasmid was transfected on the 2nd and 5th days. **G** HBV DNA replication intermediates were detected by Southern blotting. HBeAg and HBsAg levels in culture supernatants were determined by ELISA. The above experiment was independently conducted three times. Data are shown as mean ± SD. The statistical significance of the differences was assessed by the Student t test: *, P < 0.05; **, P < 0.01, ***, P < 0.001

To investigate the biological relevance of IFI35 function in human infection, we analyzed the action of IFI35 using HBV infection systems such as PHH and HepG2-NTCP cells. Pathogen-free PHHs were isolated from the tissues of four patients (donors 1, 2, 3, and 4) and the experiments were performed as shown in Fig. 4. Similar to HepG2 and Huh cells, IFI35 was mainly induced by IFN-γ in PHHs (Fig. 4A). Overexpression of IFI35 strongly inhibited HBV replication and antigens secretion in PHHs isolated from two different donors (Fig. 4C and D). To investigate whether IFI35 affects cccDNA levels, we overexpressed IFI35 and observed little effect on cccDNA levels compared to its replication inhibition. The slight decrease is probably due to the inhibition of the intra-cellular recycling pathway caused by replication inhibition (Fig. 4E). Overexpression of IFI35 in HepG2-NTCP cells also significantly inhibited viral replication and antigens secretion, similar to PHHs (Fig. 4F and G). Taken together, these results indicate that IFI35 is well induced by IFN-γ and suppresses HBV in real human hepatocytes and probably regulates at the transcriptional level of cccDNA.

### IFI35 suppresses HBV at the transcriptional level

**Fig. 5:**
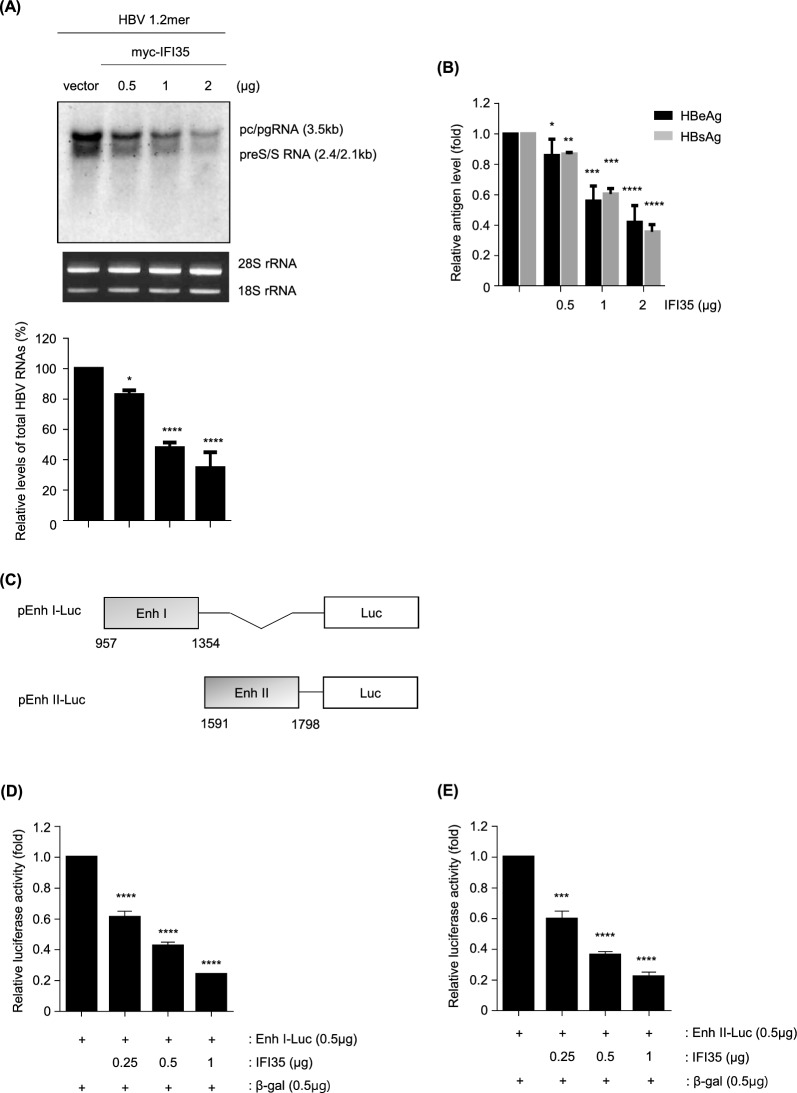
IFI35 suppresses HBV at transcriptional level. **A** HepG2 cells were co-transfected with IFI35 and HBV 1.2 plasmid. After 72 h, cells were harvested, and Northern blot analysis was conducted. 28S rRNA and 18S rRNA were loaded to confirm the quality of RNA. Relative HBV RNA levels were quantified and presented graphically (bottom). **B** HBeAg and HBsAg levels in the supernatants were determined using ELISA. **C** The illustration indicated HBV enhancer I and II reporter mutants used in this study. **D**, **E** The effect of IFI35 on each enhancer reporter was assessed in HepG2 cells. The relative luciferase activity of each enhancer clone was determined 48 h after co-transfection with either an empty vector or IFI35 vector. The total amount of transfected DNA per well was equalized by supplementing with empty vector. The above experiment was independently conducted three times. Data are shown as mean ± SD. The statistical significance of the differences was assessed by the Student t test: *, P < 0.05; **, P < 0.01, ***, P < 0.001

To determine whether IFI35 indeed regulates HBV at the transcriptional level, viral RNAs were examined after overexpression of IFI35. As shown in Fig. 5A and B, both precore/pregenomic RNAs (pc/pg RNAs) and preS/S RNAs were strongly reduced in a dose-dependent manner by IFI35, and antigens such as HBsAg and HBsAg were also significantly reduced. To assess whether IFI35 affects HBV RNA stability, HepG2 cells were co-transfected with HBV 1.2mer and either control vector or myc-tagged IFI35. After 72 h, cells were treated with Actinomycin D (5 μg/mL) to inhibit RNA polymerase activity, and HBV RNA levels were assessed by Northern blot analysis at the indicated time points. RNA decay kinetics appeared similar between control and IFI35-overexpressing cells (Supplementary Fig. 2A). Slope comparison of RNA decay curves over time showed comparable rates of RNA degradation between control and IFI35-overexpressing groups, indicating that IFI35 does not affect HBV RNA stability (Supplementary Fig. 2B). These data support that IFI35 restricts HBV at the viral RNA transcription stage. HBV transcription has been reported to be regulated by the viral enhancers and core promoter (Cp), particularly enhancer I (Enh I) and enhancer II (Enh II/Cp), which overlap with the Cp [43–46]. To determine whether IFI35 regulates the activity of viral enhancers, we constructed reporter plasmids containing each HBV enhancer (Fig. 5C) and assessed their activity with a luciferase-based reporter assay. When HepG2 cells were cotransfected with IFI35 plasmid and Enh I or Enh II/Cp reporter plasmid, respectively, the activity of the Enh I and Enh II/Cp was drastically reduced in an IFI35 dose-dependent manner, as shown in Fig. 5D and E. To further examine whether IFI35 affects other HBV promoters, we analyzed the activity of Sp1 and Sp2 luciferase reporters in the presence of increasing amounts of IFI35. HepG2 cells were co-transfected with the indicated reporter constructs together with increasing amounts of myc-IFI35. IFI35 overexpression resulted in a modest but significant reduction in luciferase activity driven by the Sp1 promoter (Supplementary Fig. 3A). Similarly, IFI35 also decreased Sp2 promoter activity in a dose-dependent manner (Supplementary Fig. 3B). However, the inhibitory effect observed for Sp1 and Sp2 was weaker than that observed for Enhancer I and Enhancer II reporters, suggesting that IFI35 primarily suppresses HBV transcription through enhancer-mediated regulation. To investigate whether the antiviral activity of IFI35 depends on HBx, HepG2 cells were transfected with either wild-type HBV 1.2mer or an HBx-deficient HBV construct HBV 1.2mer HBx– together with IFI35 plasmid. Northern blot analysis showed that overexpression of IFI35 reduced HBV transcription in both wild-type and HBx-deficient HBV 1.2mer (Supplementary Fig. 4A). Consistent with this result, ELISA analysis demonstrated that IFI35 significantly decreased the secretion of HBeAg and HBsAg under both conditions (Supplementary Fig. 4B). Southern blot analysis further revealed that IFI35 suppressed HBV replication in a dose-dependent manner regardless of the presence of HBx (Supplementary Fig. 4C). In agreement with these findings, viral antigen production was also reduced in a dose-dependent manner in both wild-type and HBx-deficient HBV (Supplementary Fig. 4D). Together, these results indicate that the antiviral activity of IFI35 against HBV replication occurs independently of HBx.

### IFI35 induce the degradation of HNF4α by ubiquitin-proteosome pathway

Hepatocyte nuclear factors (HNFs) are known to regulate HBV transcription by binding to the HBV enhancers and Cp, of which HNF1α, HNF4α and HNF3β are the most representative [47–51]. HNF1α was reported to be a proviral transcription factor that binds to the enhancer and preS1 promoter of HBV and strongly increases viral transcription [52]. HNF4α is known to be the most potent transcription factor that binds to Enh I and Enh II/Cp and increases the transcription of pc/pg RNAs [53]. On the other hand, HNF3β is known to be an antiviral transcription factor that binds to HBV enhancers in mouse NIH 3T3 fibroblasts, thereby inhibiting pgRNA synthesis [54]. Therefore, we investigated whether IFI35 regulates these transcription factors.

**Fig. 6:**
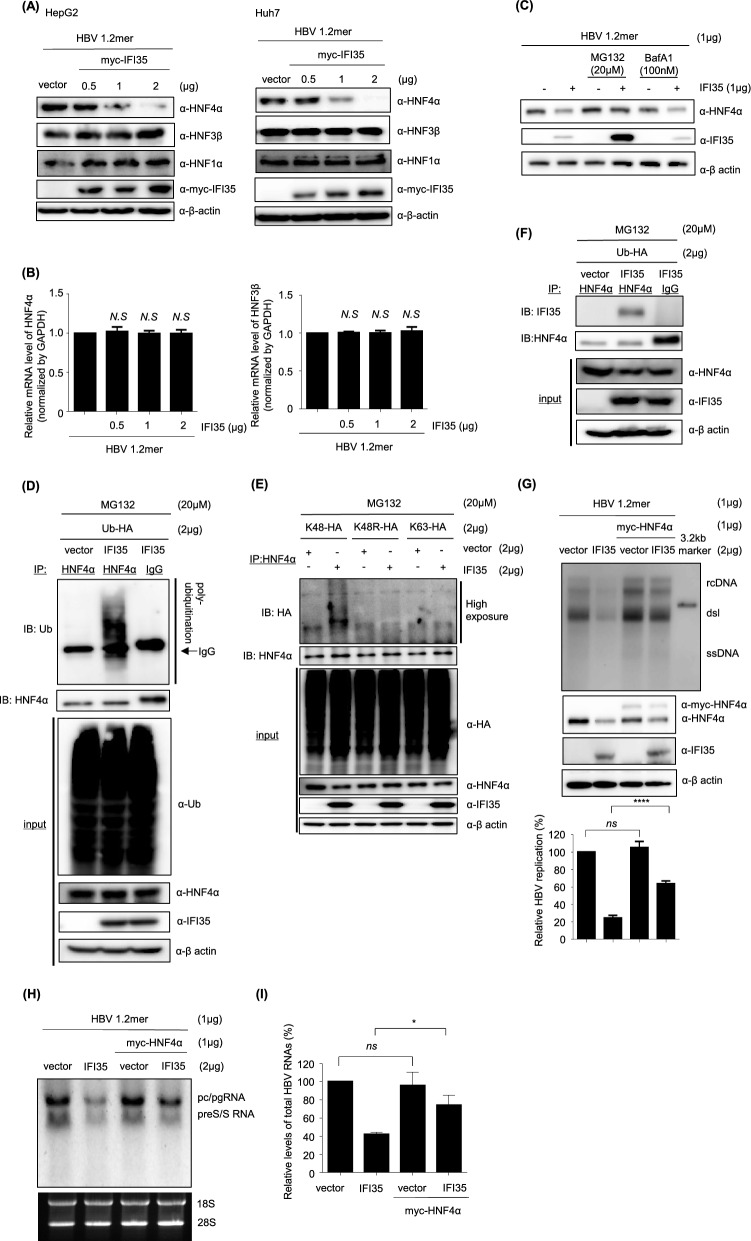
IFI35 induce the ubiquitin dependent degradation of HNF4α. **A**, **B** HepG2 and Huh7 cells were co-transfected with IFI35 and HBV 1.2 plasmid. After 72 h, the cells were lysed for Western blot analysis to confirm the protein levels of transcription factors, IFI35, and β-actin. **B** The transcription levels of HNF4α and HNF3β were quantified using real-time PCR. **C** HepG2 cells were transfected with HBV 1.2 and either myc-IFI35 plasmid or an empty vector. At 48 h after transfection, cells treated with MG132 (20 μM) and bafilomycin (100 nM) were collected to measure the level of HNF4α. β-actin was used as a loading control. **D** HepG2 cells were co-transfected with plasmids expressing Ubiquitin-HA and either myc-IFI35 or an empty vector. At 48 h after transfection, MG132 (20 μM) was treated for 6 h. Cell lysates were prepared and immunoprecipitated using anti-HNF4α antibody. **E** HepG2 cells were co-transfected with plasmids encoding HA-tagged ubiquitin constructs: K48-only, K48R (lysine 48 to arginine mutant), or K63-only, along with either control vector or myc-IFI35. At 48 h of post-transfection, cells were treated with MG132 for 6 h. Total cell lysates were subjected to immunoprecipitation using an anti-HNF4α antibody, followed by immunoblotting with an anti-HA antibody to detect ubiquitinated HNF4α. Total protein levels of HNF4α, IFI35, and HA-tagged ubiquitin in input lysates were analyzed by immunoblotting. **F** HNF4α interacts with IFI35. Cell lysates were precipitated with an anti-HNF4α antibody and subsequently subjected to immunoblotting. **G** The effect of HNF4α on IFI35-mediated inhibition of HBV replication was evaluated. Viral replication and protein expression were determined through Southern blot and Western blot analyses, respectively. **H** HepG2 cells were co-transfected with HBV 1.2mer (1 μg), Myc-HNF4α (1 μg), and Myc-IFI35 (2 μg) as indicated. At 72 h post-transfection, total RNA was analyzed by Northern blotting to detect HBV transcription. **I** Relative levels of total HBV RNAs from **H** were quantified by densitometric analysis and normalized to rRNA. The above experiment was independently conducted three times. Data are shown as mean ± SD. The statistical significance of the differences was assessed by the Student t test: *, P < 0.05; **, P < 0.01, ***, P < 0.001

As shown in Fig. 6A, among the three HNFs, only HNF4α was dose-dependently reduced by IFI35 in both HepG2 and Huh7 cells. To determine whether IFI35 inhibits the transcription of HNFs, we measured the mRNA levels of HNF4α and HNF3b by real-time-PCR. However, IFI35 did not change the mRNA levels of HNF4α and HNF3β (Fig. 6B). To determine by which pathway IFI35-induced HNF4α degradation occurs, we examined the amount of HNF4α in HepG2 cells after treatment with MG132, a proteasome inhibitor, or BafA1, which inhibits autophagic degradation. Western blotting showed that the degradation of HNF4α was restored by MG132 but not by BafA1 (Fig. 6C). Therefore, we performed a ubiquitination assay and found that IFI35 promoted the ubiquitination of HNF4α. (Fig. 6D). To determine which lysine residues of ubiquitin are involved in HNF4α degradation, we performed a ubiquitination assay after transfection with HA-tagged ubiquitin constructs that selectively form K48-linked, K48R-mutant (lysine 48 to arginine), or K63-linked ubiquitin chains. Upon IFI35 overexpression, a substantial increase in HNF4α polyubiquitination was observed exclusively in the presence of K48-linked ubiquitin, but not with K63-linked or K48R constructs. These results indicate that IFI35 facilitates HNF4α degradation predominantly through K48-linked polyubiquitination (Fig. 6E). Immunoprecipitation analysis showed that HNF4α interacts with IFI35 (Fig. 6F). These results suggest that the degradation of HNF4α by IFI35 is mediated through the ubiquitin–proteasome pathway in which the interaction between IFI35 and HNF4α may play a role. To verify whether IFI35-induced reduction of HNF4α is the primary mechanism of our findings, we supplemented HNF4α after overexpression of IFI35. The HBV replication inhibited by IFI35 was significantly restored by the supply of HNF4α (Fig. 6G). Northern blot analysis showed that IFI35 markedly reduced HBV transcription (Fig. 6H,I). Supplementation of HNF4α partially restored HBV RNA levels suppressed by IFI35, consistent with the restoration of HBV replication intermediates observed in the Southern blot. To examine whether the antiviral activity of IFI35 is mediated through HNF4α, HepG2 cells were co-transfected with HBV 1.2mer together with either control siRNA or siRNA targeting HNF4α, followed by IFI35 overexpression. As expected, knockdown of HNF4α markedly reduced HBV replication in southern blotting, consistent with the essential role of HNF4α in HBV transcription and replication (Supplementary Fig. 5A). Under these conditions, HBV replication levels were already strongly suppressed by HNF4α depletion, making it difficult to directly compare the additional effect of IFI35 overexpression on HBV replication. We therefore assessed viral antigen production by ELISA. Consistent with the Southern blot results, IFI35 overexpression significantly reduced the secretion of HBeAg and HBsAg in control cells (Supplementary Fig. 5B). In contrast, under HNF4α-depleted conditions, IFI35 overexpression did not further reduce antigen production. These results suggest that the antiviral activity of IFI35 is largely mediated through targeting HNF4α. These results suggest that IFI35 inhibits HBV replication primarily through degradation of HNF4α.

### TRIM21 is required for IFI35-induced ubiquitination of HNF4α

**Fig. 7:**
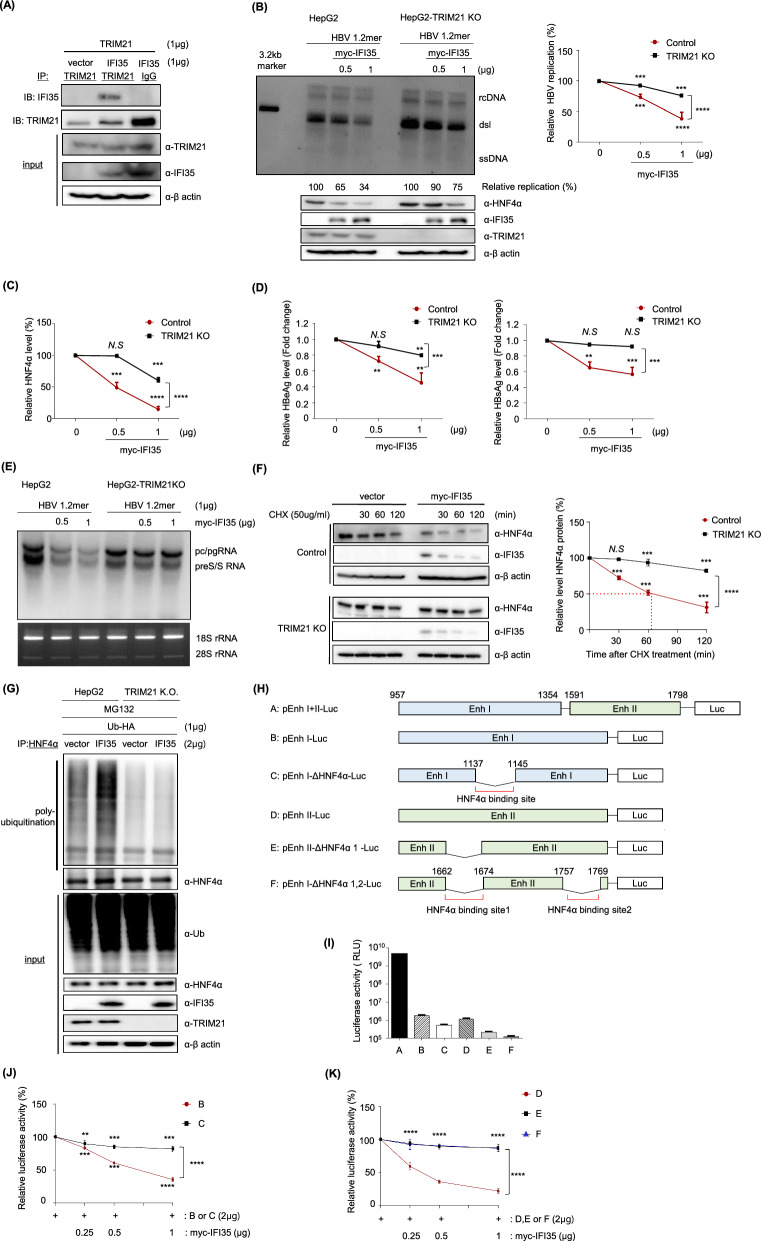
IFI35 ubiquitinates HNF4a in TRIM21-dependent manner. **A** Cell lysates were immunoprecipitated using an anti-TRIM21 antibody. **B** Detection of HBV DNAs by Southern blot from HepG2 and HepG2-TRIM21-KO cells. Expression levels of HNF4α, IFI35 and TRIM21 in HepG2-TRIM21-KO cells compared to parental HepG2 cells (Control) were assessed by Western blot. HBV DNA levels of HepG2 and HepG2-TRIM21-KO cells were quantified in graph (bottom). **C** Quantification of HNF4α protein levels in HepG2 and HepG2-TRIM21 knockout (KO) cells transfected with increasing amounts of myc-IFI35. HNF4α levels were determined by immunoblotting and quantified relative to the vector control. Data represent mean ± SD from three independent experiments. **D** Secreted HBeAg and HBsAg levels were measured by ELISA in HepG2 and HepG2-TRIM21-KO cells transfected with increasing amounts of myc-IFI35. Data represent mean ± SD from three independent experiments. **E** HepG2 or HepG2-TRIM21 knockout cells were transfected with HBV 1.2mer together with increasing amounts of IFI35. Total RNA was analyzed by Northern blotting to detect HBV transcription. **F** Effect of TRIM21 on IFI35-mediated HNF4α degradation. HepG2 and HepG2-TRIM21-KO cells were transfected with either empty vector or myc-IFI35. After 48 h, cycloheximide (50ug/ml) were treated for the indicated times, and cells were harvested. Expression levels of HNF4α and IFI35 were evaluated through Western blotting. Quantification of HNF4α levels between HepG2 and HepG2-TRIM21-KO cells (bottom). **G** HepG2 cells and TRIM21 knockout cells were co-transfected with HA-tagged ubiquitin (1 μg) and either vector or IFI35 (2 μg). At 48 h post-transfection, HNF4α was immunoprecipitated and immunoblotted with anti-ubiquitin antibody to assess polyubiquitination. Total levels of HNF4α, IFI35, TRIM21, and HA-tagged ubiquitin in input lysates were also analyzed by immunoblotting. **H** Schematic diagram of HBV enhancer luciferase reporter constructs. Constructs containing enhancer I (EnhI), enhancer II (EnhII), or both regions were cloned upstream of the luciferase reporter gene. Mutant constructs lacking HNF4α binding sites were generated in EnhI (ΔHNF4α) or EnhII (ΔHNF4α1 and ΔHNF4α1,2) as indicated. **I** Basal luciferase activity of HBV enhancer reporter constructs. HepG2 cells were transfected with the indicated luciferase reporter plasmids, and luciferase activity was measured. **J** Comparison of the effect of IFI35 on EnhI-driven transcription with or without the HNF4α binding site. HepG2 cells were co-transfected with the indicated EnhI luciferase reporters and increasing amounts of myc-IFI35. Luciferase activity was measured 48 h post-transfection. Data represent mean ± SD from three independent experiments. **K** Comparison of the effect of IFI35 on EnhII-driven transcription with or without HNF4α binding sites. HepG2 cells were co-transfected with EnhII luciferase reporters containing wild-type or mutated HNF4α binding sites together with increasing amounts of myc-IFI35. Luciferase activity was measured 48 h post-transfection. Data represent mean ± SD from three independent experiments. The above experiment was independently conducted three times. Data are shown as mean ± SD. The statistical significance of the differences was assessed by the Student t test: *, P < 0.05; **, P < 0.01, ***, P < 0.001

Based on the results so far, it is not known whether IFI35 directly mediates K48-linked ubiquitination of HNF4α or facilitates the recruitment of any E3 ubiquitin ligase. However, since IFI35 lacks typical E3 ubiquitin ligase domains such as HECT, RING, or F-box motifs [55], it is likely that IFI35 functions as an adaptor, recruiting specific E3 ligase for HNF4α ubiquitination. A previous study reported that TRIM21 mediates the ubiquitination of N-myc and STAT interacting protein (Nmi) to form a stable Nmi-IFI35 complex and promote the negative regulatory function of this complex on the innate antiviral response [21]. To determine whether IFI35 recruits TRIM21 to ubiquitinate HNF4α, the interaction between TRIM21 and IFI35 was examined by immunoprecipitation following transient expression of TRIM21 and IFI35 in HepG2 cells. This experiment confirmed the interaction between IFI35 and TRIM21 in hepatocytes (Fig. 7A). To further elucidate the role of TRIM21 in IFI35-mediated HNF4α degradation, we generated a TRIM21 knockout HepG2 cell line (HepG2-TRIM 21 (KO) and then analyzed the inhibition of HBV replication by IFI35 compared to that in the HepG2 cells. Investigating the levels of HBV replication and HNF4α by Southern blotting (Fig. 7B) and Western blotting (Fig. 7C), respectively, we found that their reduction by IFI35 was significantly attenuated in TRIM21-deficient HepG2 cells. Similarly, the level of HBeAg and HBsAg in TRIM21 KO HepG2 cells were mostly not significantly reduced by IFI35 compared to those in control cells (Fig. 7D). We next examined whether TRIM21 is required for the transcriptional suppression of HBV by IFI35. In wild-type HepG2 cells, IFI35 overexpression significantly reduced HBV transcripts. In contrast, in HepG2-TRIM21 knockout cells, IFI35 failed to efficiently suppress HBV RNA levels (Fig. 7E). These results indicate that IFI35-mediated repression of HBV transcription requires TRIM21.

The stability of HNF4α protein after treatment with cycloheximide (CHX), a translation inhibitor, was compared in HepG2-TRIM21 KO and HepG2 cells (Fig. 7F). The CHX chase assay showed that the stability of HNF4α in HepG2 cells was rapidly reduced by IFI35, resulting in a half-life of less than 60 min, while in the TRIM21 KO cells, the stability was only slightly reduced even after 2 h, indicating a very long half-life (Fig. 7F, right). To further determine whether IFI35 promotes HNF4α ubiquitination through TRIM21, we compared HNF4α ubiquitination levels in wild-type HepG2 cells and TRIM21 knockout (KO) cells. Cells were co-transfected with IFI35 and HA-tagged ubiquitin, followed by treatment with MG132 to prevent proteasomal degradation. In HepG2 cells, overexpression of IFI35 markedly increased the polyubiquitination of HNF4α (Fig. 7G). In contrast, in TRIM21 KO cells, IFI35 overexpression failed to enhance HNF4α ubiquitination. These results indicate that IFI35-mediated ubiquitination of HNF4α is dependent on TRIM21, supporting the model in which IFI35 recruits TRIM21 to promote ubiquitination and degradation of HNF4α. These results indicate that the degradation of HNF4α by IFI35 requires TRIM21.

HBV transcription is regulated by two enhancer regions, Enhancer I (EnhI) and Enhancer II (EnhII)[56], which are illustrated schematically in Fig. 7H. To further examine whether IFI35 affects HBV transcription through HNF4α-dependent regulation, we generated luciferase reporter constructs containing either EnhI or EnhII with deletions of HNF4α binding sites. HepG2 cells were transfected with each construct at equal amounts, and luciferase activity was measured. The construct containing both EnhI and EnhII exhibited the highest transcriptional activity (Fig. 7I). Reporter constructs containing EnhI alone showed substantially reduced activity compared with the EnhI + II construct, and deletion of the HNF4α binding site within EnhI further decreased luciferase activity. Similarly, the EnhII reporter displayed measurable transcriptional activity, which was progressively reduced upon deletion of HNF4α binding sites. Removal of one HNF4α binding site in EnhII significantly decreased luciferase activity, whereas deletion of both sites nearly abolished reporter activity. These results indicate that HNF4α binding sites within EnhI and EnhII are critical for HBV transcriptional activation. To determine whether IFI35 suppresses HBV transcription through HNF4α-dependent enhancer activity, we examined the effect of IFI35 on EnhI and EnhII luciferase reporter constructs containing wild-type or mutated HNF4α binding sites. For the EnhI reporter, co-transfection of IFI35 resulted in a dose-dependent decrease in luciferase activity (Fig. 7J). However, deletion of the HNF4α binding site in EnhI significantly attenuated the inhibitory effect of IFI35, indicating that IFI35-mediated suppression of EnhI activity largely depends on the presence of the HNF4α binding site. Similarly, IFI35 overexpression reduced luciferase activity driven by the EnhII reporter in a dose-dependent manner (Fig. 7K). In contrast, deletion of one or both HNF4α binding sites in EnhII markedly diminished the inhibitory effect of IFI35. These results indicate that IFI35 suppresses HBV transcription through inhibition of HNF4α-dependent enhancer activity. All these results indicate that IFI35 inhibits HBV transcription and replication by promoting the degradation of HNF4α through the TRIM21-mediated ubiquitin-proteosome pathway.

### Domain mapping of IFI35 identifies NID domains as mediators of TRIM21–HNF4α complex formation

**Fig. 8:**
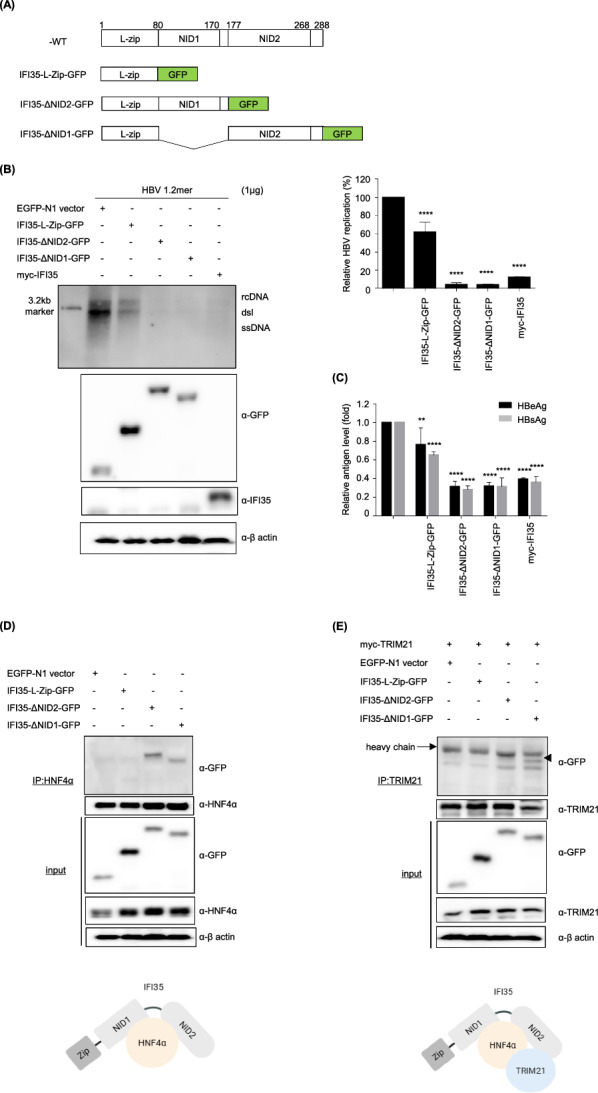
Domain mapping of IFI35 as mediators of TRIM21–HNF4α complex formation. **A** Schematic representation of IFI35 constructs used in this study. GFP-tagged mutants lacking NID1 (ΔNID1) or NID2 (ΔNID2), as well as the leucine zipper construct (Zip), were generated. **B** HepG2 cells were co-transfected with HBV 1.2mer and the indicated IFI35 constructs. At 72 h post-transfection, HBV replication intermediates were analyzed by Southern blotting. Quantification of HBV replication levels based on Southern blot analysis. **C** Secreted HBeAg and HBsAg levels in the culture supernatants were measured by ELISA. **E** Co-immunoprecipitation analysis of IFI35 mutants with HNF4α. HepG2 cells were transfected with GFP-tagged IFI35 constructs and cell lysates were immunoprecipitated using anti-HNF4α antibody followed by immunoblotting with anti-GFP antibody. **F** Interaction of IFI35 mutants with TRIM21. Cells were transfected with Myc-TRIM21 together with the indicated IFI35 constructs. Cell lysates were subjected to immunoprecipitation using anti-TRIM21 antibody followed by immunoblotting with anti-GFP antibody. Data represent mean ± SD (n = 3). Statistical significance was determined relative to the vector control

IFI35 contains three major domains: an N-terminal leucine zipper (Zip) followed by two tandem N-myc–interacting domains (NID1 and NID2). To define the functional domains of IFI35 required for its antiviral activity against HBV and its interaction with host factors, we generated three mutant constructs: a Zip-only construct and deletion mutants lacking either NID1 or NID2, based on previously reported studies [57] (Fig. 8A). HepG2 cells were co-transfected with HBV 1.2mer together with GFP-tagged IFI35 constructs, and HBV replication and antigen secretion were analyzed. IFI35 mutants lacking either NID1 or NID2 retained antiviral activity and significantly suppressed HBV replication, whereas the construct containing only the leucine zipper domain (Zip) showed minimal inhibitory effect (Fig. 8B). Consistent with these findings, ELISA analysis demonstrated that both ΔNID1 and ΔNID2 mutants significantly reduced the secretion of HBeAg and HBsAg, comparable to full-length IFI35 (Fig. 8C). In contrast, loss of both NID domains abolished the antiviral activity, indicating that the presence of at least one NID domain is required for IFI35-mediated suppression of HBV replication. To investigate which domains are responsible for interaction with HNF4α and TRIM21, we next performed co-immunoprecipitation analyses. IFI35 mutants retained interaction with HNF4α, indicating that the NID domains contribute to the association between IFI35 and HNF4α (Fig. 8D). In contrast, deletion of the NID2 domain markedly impaired the interaction between IFI35 and TRIM21, whereas the ΔNID1 mutant maintained this interaction (Fig. 8E). Given that TRIM21 has previously been reported to directly interact with HNF4α [37], these findings suggest that IFI35 functions as a scaffold protein that bridges TRIM21 to HNF4α through its NID domains. In this model, IFI35 binds HNF4α via the NID domains while recruiting TRIM21 through the NID2 domain, thereby facilitating TRIM21-mediated ubiquitination and degradation of HNF4α to suppress HBV replication (Fig. 8F).

### IFI35 potently suppresses HBV in an in vivo mouse model

**Fig. 9:**
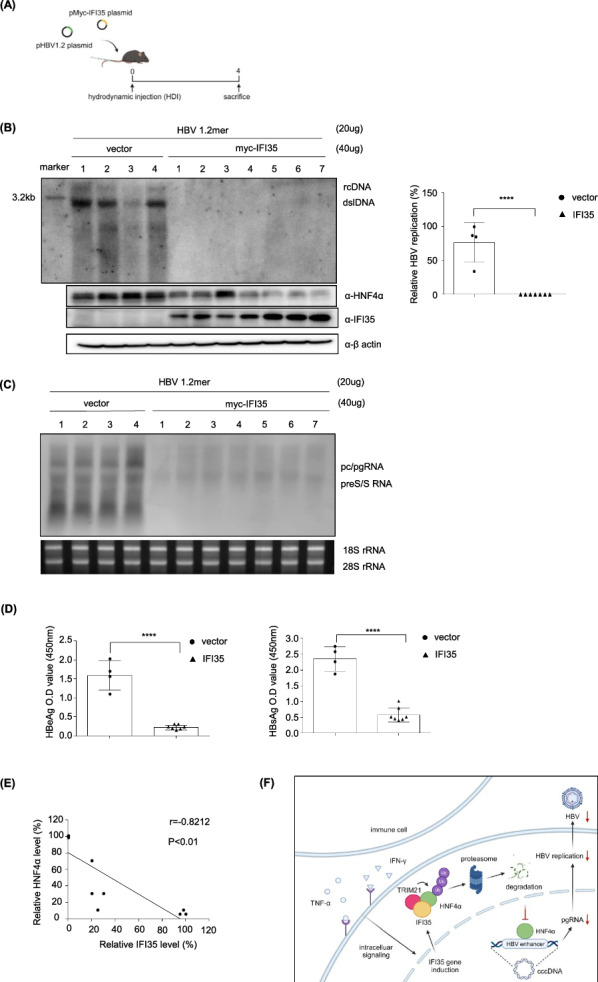
IFI35 downregulates HBV replication in in vivo mouse model. **A** Schematic of study design. Mice were hydrodynamically injected with HBV 1.2 and either empty vector or myc-IFI35 plasmids. Mice were sacrificed at 7 days post-injection. **B** Intrahepatic HBV DNA levels at 7 days post-injection were assessed by Southern blot. Western blot analysis was performed to determine HNF4α and IFI35 expression levels. **C** Total RNA extracted from liver tissues of hydrodynamically injected HBV mouse models was analyzed by Northern blotting. The 18S and 28S rRNA bands are shown as loading controls. **D** HBeAg and HBsAg levels in mouse serum were measured by ELISA. **E** Quantification reveals the correlation between HNF4α and IFI35 expression levels. Data are shown as mean ± SD. The statistical significance of the differences was assessed by the Student t test: *, P < 0.05; **, P < 0.01, ***, P < 0.001

Finally, we evaluated the anti-HBV efficacy of IFI35 using a hydrodynamically injected HBV mouse model (Fig. 9A). Southern blot analysis showed a dramatic reduction in HBV replication in liver tissues of all seven mice hydrodynamically injected with IFI35 compared to the control group (Fig. 9B). Consistent with the reduction in HBV replication observed by Southern blot analysis, Northern blot analysis of the same liver samples revealed a marked decrease in HBV transcription in mice expressing IFI35 compared with the control group (Fig. 9C). Consistent with the level of viral replication, IFI35 significantly reduced HBV antigens (HBeAg and HBsAg) in mouse sera (Fig. 9D). Very interestingly, IFI35 also significantly reduced mouse HNF4α, with an exact inverse correlation (p < 0.0001) between the protein levels of IFI35 and HNF4α. (Fig. 9B and E). Furthermore, as shown in Supplementary Fig. 6C correlation analyses in three HBV-related patient datasets (GSE83148, GSE96851, and GSE14668) revealed a consistent and statistically significant inverse relationship between IFI35 and HNF4α expression. These results demonstrate that IFI35 inhibits HBV replication in vivo by effectively reducing HNF4α protein.

## Discussion

In this study, we identified IFI35 as a novel anti-HBV protein that potently inhibits HBV replication by proteomic analysis of hepatocytes treated with TNF-α and IFN-γ, which are known to be representative antiviral cytokines. The IFI35 was mainly induced by IFN-γ and inhibited the transcription of HBV cccDNA. Mechanistically, IFI35 recruited the E3 ligase TRIM21 to promote the degradation of HNF4α, a transcription factor essential for HBV transcription (Fig. 9E). In PHH and animal studies, IFI35 showed potent suppressive activity against HBV.

The host innate immune systems have evolved complicated mechanisms for defending against viral infections. Numerous studies have led to an understanding of the different mechanisms of IFN-induced signaling in how cells detect viruses, how they effectively defend against viruses, and how they completely clear viruses from infected cells. In HBV infection, the host can induce many IFN-stimulated genes (ISGs) to regulate IFN signaling and suppress viral infection [58]. In studies using HBV transgenic mice, HBV-specific cytotoxic T lymphocytes (CTLs) can inhibit HBV gene expression and replication without hepatocyte killing, primarily by increasing TNF-α production, which is known as a noncytolytic mechanism [59, 60]. The APOBEC family, which is induced in hepatocytes by TNF-α and IFN-γ produced by T cells, exhibits antiviral activity against HBV, of which APOBEC3A and APOBEC3B in particular can reduce the level of HBV cccDNA [14]. Another ISG, ISG20, can inhibit viral replication by selectively targeting and degrading N6-methyladenosine modified HBV RNAs, and ISG20 also collaborates with APOBEC3A to degrade deaminated cccDNA [61, 62]. IFN-γ-induced indoleamine-2,3-dioxygenase (IDO) also efficiently reduces HBV DNA levels and protein synthesis [63, 64]. Despite these diverse mechanisms, there is still a need to study more detailed immune mechanisms to understand which ISGs are mediated by IFN-γ and TNF-α and how they inhibit HBV.

The multiple functions of IFI35 in viral infections have been explored in several studies. IFI35 has been shown to either promote or inhibit the type I IFN response to a diverse range of viral infections, such as foot-and-mouth disease virus (FMDV), vesicular stomatitis virus (VSV), and bovine foamy virus (BFV) [23–25]. IFI35 leads to the downregulation of the host antiviral response. RIG-I senses viral infections, such as VSV and Sendai virus (SeV), and triggers the induction of ISGs[65]. During this process, IFI35 interacts with RIG-I to inhibit its activation by promoting K48-linked ubiquitination and subsequent proteasomal degradation [24]. The specific E3 ubiquitin ligase involved in this process is not yet identified. In influenza A virus (IAV) infection, IFI35 modulates RIG-I-mediated antiviral signaling by binding to IAV NS1 protein. Interestingly, NS1 of the H3N2 virus interacts with IFI35 and effectively inhibits IFI35-dependent ubiquitination of RIG-I, whereas H7N9 NS1 does not [57]. Upon infection with VSV or SeV, the innate antiviral pathways activate the production of IFN-α/β [66], which in turn leads to the induction of many ISGs such as IFI35 and Nmi. Induced IFI35 interacts with Nmi [17], which then recruits TRIM21 to ubiquitinate Nmi, primarily through K63-linked ubiquitination. This facilitates the interaction between Nmi and IFI35 and consequently leads to the suppression of IFN-β production [21].

As shown by the aforementioned studies, IFI35 is closely associated with ubiquitination of host proteins during viral infection, and the mechanisms differ depending on the virus types. Our study highlights the involvement of TRIM21 in IFI35 mediated K48-ubiquitination of HNF4α, leading to the degradation of HNF4α. However, further studies are needed to determine whether IFI35 interacts with other E3 ligases for its antiviral activity, or whether it may have other activities associated with K63-ubiquitination.

Previous studies have shown that HNF4α can be regulated by various signaling pathways or host factors, or its subcellular distribution can be altered during HBV infection. Our previous studies have shown that interleukin-32 and cleaved c-FLIP can downregulate HNF4α expression via MAPK kinase-1/2 signaling pathways [30, 31]. Steroid receptor coactivator 3 (SRC-3) has been reported to inhibit HBV gene expression by activating Akt signaling to prevent HNF4α nuclear translocation [67]. Estrogen receptor (ER)-α affects the transcription of HBV by blocking the binding of HNF-4α to HBV Enh I and Enh II. ER-α translocated into the nucleus binds to HNF-4α and prevents HNF4α from binding to the HBV Enh I and Enh II, thereby decreasing HBV transcription [68]. In addition, the regulation of HNF4α expression is dependent on interaction between transcription factors such as HNF1β, SP1, and HNF6 [69]. These previous studies indicate that HNF4α is essential for HBV RNA transcription, so the host can easily control viral replication efficiently and robustly by regulating its amount or cellular distribution. Therefore, from the perspective of the host's immune system, HNF4α may be an excellent target for controlling HBV, as shown in our study and other previous studies. Here, we have identified a novel mechanism for IFI35-mediated degradation of HNF4α. However, we cannot exclude the possibility that IFI35 affects HBV replication by other unknown mechanisms, as replication of HBV was not completely restored when HNF4α was supplemented (Fig. 6G).

When we analyzed public datasets using human liver tissues at various clinical stages, we found that IFI35 levels were elevated in patients with chronic HBV infection and HBV-associated liver failures (Fig. 1E). This may be due to the induction of IFI35 by increased antiviral cytokines secreted by CTLs or other immune cells due to chronic HBV infection and HBV-associated liver failures. Interestingly, however, IFI35 was rather reduced in patients treated with NUC antiviral agents (Fig. 1E). This is probably due to the decreased secretion of antiviral cytokines such as IFN-γ and TNF-α by CTLs due to decreased viral load.

Chronic hepatitis B infection is clinically categorized into four phases based on serum HBV DNA levels, HBeAg status, and alanine aminotransferase (ALT) levels: immunotolerant (IT), immune-active (IA), inactive carrier (IC), and HBeAg-negative hepatitis (ENEG) [70]. IFI35 expression progressively decreased across these phases in parallel with the reduction of serum HBV DNA levels, suggesting a positive association between viral replication and IFI35 induction (Supplementary Fig. 6A). In public transcriptomic datasets (GSE83148, GSE96851, and GSE14668), IFI35 expression was significantly increased in HBV-infected patients compared to healthy controls, whereas HNF4α expression was consistently downregulated (Supplementary Fig. 6B). Correlation analysis revealed a significant negative correlation between IFI35 and HNF4α expression across all three datasets, indicating that IFI35-mediated downregulation of HNF4α may be clinically relevant in HBV infection (Supplementary Fig. 6C). As IFI35 is an interferon-inducible protein, IFN therapy alters IFI35 expression in patients. In GSE84346, IFI35 expression was significantly upregulated in pegylated-IFN-α–treated HCV-infected patients compared to untreated HCV-infected patients (Supplementary Fig. 6D, left panel). This finding is consistent with previous reports showing IFI35 protein induction by IFN-α in HT29 cells [17]). Moreover, in GSE24427, which includes blood samples from multiple sclerosis patients undergoing IFN-β therapy, IFI35 expression was increased at 1, 12, and 24 months post-injection (Supplementary Fig. 6D, right panel), which was consistent with prior studies reporting IFN-β–induced IFI35 upregulation in human mesangial cells [71]. All of these studies further support clinical relevance of our findings.

In summary, we identified IFI35 as a novel protein that strongly reduces HBV transcription and replication. In the future, the IFI35-TRIM21-HNF4α axis may be a potential target for the suppression of HBV.

## Conclusions

While antiviral cytokines such as TNF-α and IFN-γ are known to inhibit HBV replication, the host cellular factors mediating these effects remain largely unidentified. Our findings demonstrate that IFI35 suppresses HBV enhancer activity by promoting ubiquitin-mediated proteasomal degradation of HNF4α, a key transcription factor essential for HBV transcription. Moreover, IFI35 recruits tripartite motif-containing protein 21 (TRIM21), an E3 ubiquitin ligase, to facilitate K48-linked ubiquitination of HNF4α. Collectively, these results identify IFI35 as a novel host protein that significantly restricts HBV transcription and replication. The IFI35–TRIM21–HNF4α axis thus represents a potential therapeutic target for the suppression of HBV infection.

## Supplementary Information

Supplementary file 1.

Supplementary file 2. Figure 1. IFN-γ strongly induces IFI35 expression and reduces HBV transcription in HepG2 cells. (A) HepG2 cells were transfected with HBV 1.2mer (1μg) and subsequently treated with concentrations of IFN-α or IFN-γ (100, 500, and 1000 U/ml). Whole-cell lysates were collected and analyzed by immunoblotting using the indicated antibodies. ISG20 was included as a representative interferon-stimulated gene to confirm activation of interferon signaling pathways. β-actin served as a loading control. Representative immunoblots from three independent experiments (n = 3) are shown. HepG2 cells were transfected with HBV 1.2mer (1 μg). 6 hours post-transfection, cells were treated with IFN-γ at the indicated concentrations (250, 500, and 1000 U/ml) for three times, and harvested at 72 h post-transfection. (B) HBV transcripts (pgRNA and preS/S RNA) were analyzed by Northern blotting; 18S and 28S rRNA are shown as loading controls. (C) Secreted HBeAg and HBsAg in culture supernatants were quantified and presented as relative levels normalized to the untreated control. Data represent mean ± SD (n = 3). Statistical significance was determined relative to the untreated control (*p < 0.05, ***p < 0.001, ****p < 0.0001). Figure 2. Effect of IFI35 on HBV RNA stability. (A) HepG2 cells were transfected with either control vector or IFI35 expression plasmid. At 72 hours post-transfection, cells were treated with Actinomycin D (10 μg/mL) and total RNA was harvested at 0, 2, 4, and 6 hours post treatment. HBV RNA levels were assessed by Northern blotting. Ethidium bromide staining of 28S and 18S rRNA served as loading controls. (B) Quantification of HBV RNA decay from (A) is shown. RNA degradation patterns were compared between control and IFI35-overexpressing cells following Actinomycin D treatment. Figure 3. Effect of IFI35 on SP1 and SP2 promoter activity. (A) Schematic representations of the preS1p-Luc and preS2p-Luc luciferase reporter constructs are shown. (B and C) HepG2 cells were co-transfected with 2μg of either preS1p-Luc (B) or preS2p-Luc (C) reporter plasmid, along with 0.5 μg of β-galactosidase plasmid as a loading control. Increasing amounts of IFI35 expression plasmid (0.25, 0.5, or 1 μg) were co-transfected. Luciferase activity was measured 48 hours post-transfection and normalized to β-galactosidase activity. The above experiment was independently conducted three times. Data are presented as mean ± standard deviation (SD). Statistical significance was assessed by Student’s *t*-test: *, P < 0.05; **, P < 0.01, ***; P < 0.001. Figure 4. IFI35 suppresses HBV replication independent of HBx. (A) HepG2 cells were co-transfected with wild-type HBV 1.2mer or HBx-deficient HBV (HBV 1.2mer HBx–) together with vector or IFI35 expression plasmid. HBV transcription was analyzed by Northern blotting. Ribosomal RNA (18S and 28S) was used as a loading control. (B) Secreted HBeAg and HBsAg levels in culture supernatants were measured by ELISA. (C) HepG2 cells were transfected with HBV 1.2mer or HBV 1.2mer HBx– together with increasing amounts of IFI35. HBV replication intermediates were analyzed by Southern blotting. (D) Quantification of secreted HBeAg and HBsAg levels following IFI35 overexpression. Figure 5. Effect of HNF4α knockdown on IFI35-mediated suppression of HBV replication. (A) HepG2 cells were transfected with HBV 1.2mer (1 μg) together with control siRNA or siRNA targeting HNF4α (10 nM). Cells were additionally transfected with either empty vector or Myc-IFI35 expression plasmid (1 μg) as indicated. HBV replication intermediates were analyzed by Southern blotting. Protein expression levels of HNF4α and IFI35 were confirmed by immunoblotting. β-actin served as a loading control. Representative results from three independent experiments (n = 3) are shown. (B) The levels of secreted HBeAg and HBsAg in the culture supernatant were measured and normalized to the vector control. IFI35 overexpression reduced both HBeAg and HBsAg levels, whereas HNF4α knockdown markedly suppressed viral antigen production. Data represent mean ± SD (n = 3). Statistical significance was determined relative to the corresponding vector control (**p < 0.01, ****p < 0.0001). Figure 6. Analysis of public datasets for IFI35 and HNF4α expression. (A) Analysis of IFI35 expression levels across different clinical phases of HBV infection based on publicly available transcriptomic data. (B) HNF4α expression levels were examined in HBV-infected patients and healthy controls using datasets GSE83148, GSE96851, and GSE14668. (C) Correlation analysis between IFI35 and HNF4α expression in the same datasets. (D) IFI35 expression was analyzed in patients undergoing interferon (IFN) therapy using GSE84346 (peg-IFN-α–treated HCV-infected patients) and GSE24427 (IFN-β–treated multiple sclerosis patients). Statistical significance was assessed by Student’s t-test: *, P < 0.05; **, P < 0.01; ***, P < 0.001

Supplementary file 3.

## Data Availability

The datasets used and analyzed during the current study are available from the corresponding author upon reasonable request.
