# Supplementary material for: IFI35 suppresses the transcription of hepatitis B virus cccDNA minichromosome via promoting HNF4α proteasomal degradation
Source: J Biomed Sci. 2026 Mar 30;33:36. doi: 10.1186/s12929-026-01239-w (PMC13034610; doi:10.1186/s12929-026-01239-w)

Supplementary fig 1.

(A)

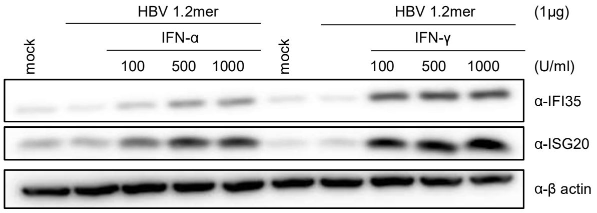

(B)

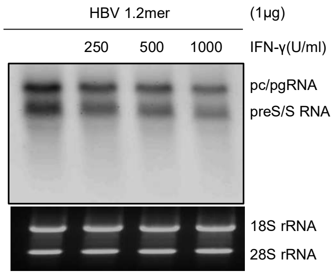

(C)

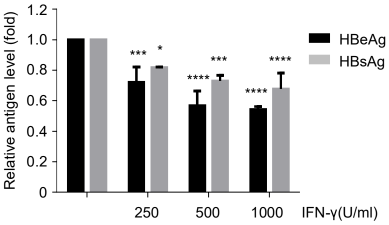

Supplementary fig 2.

(A)

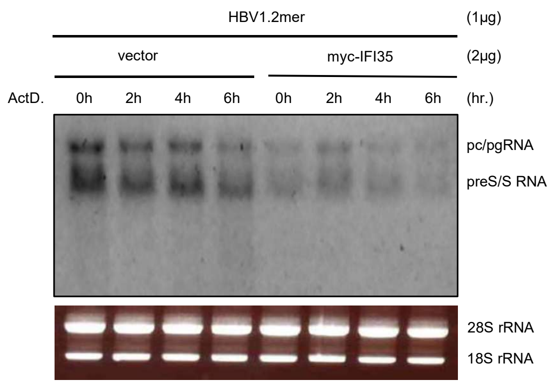

(B)

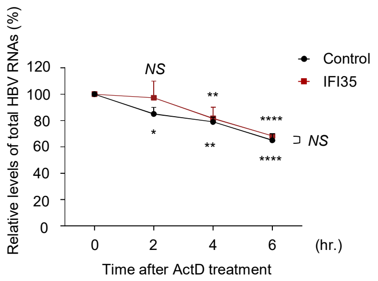

Supplementary fig 3.

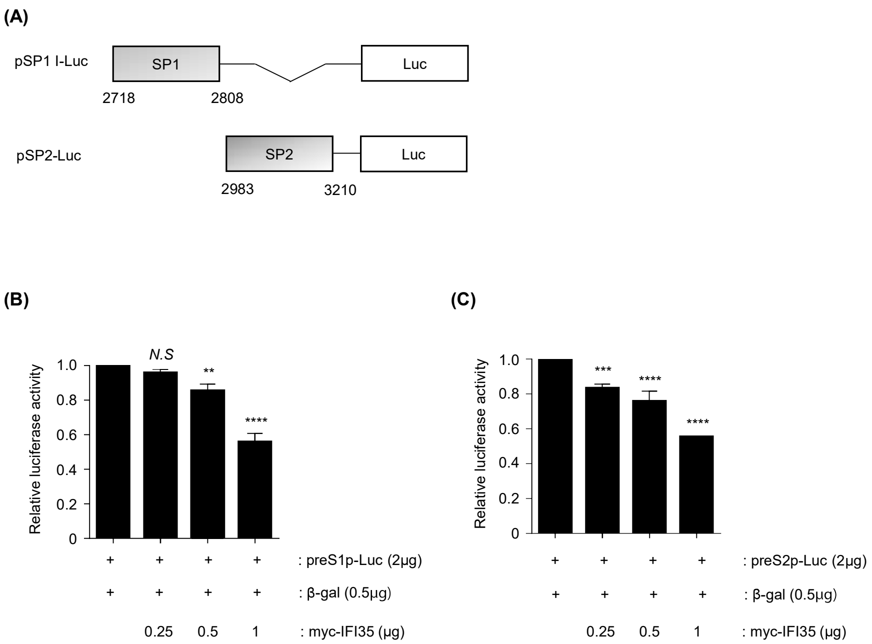

Supplementary fig 4.

(A)

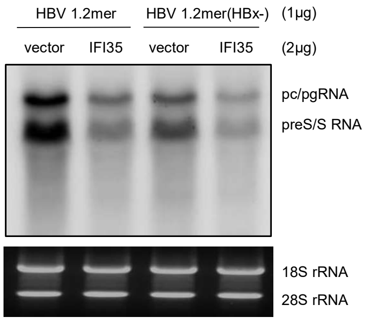

(B)

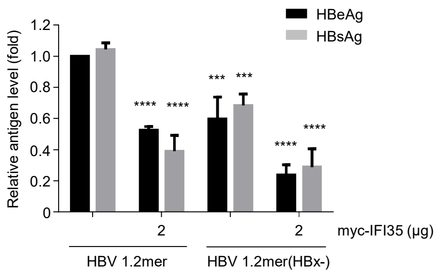

(C)

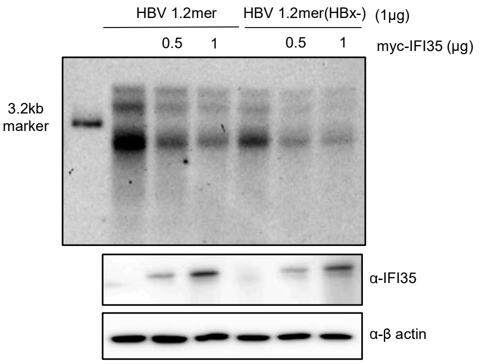

(D)

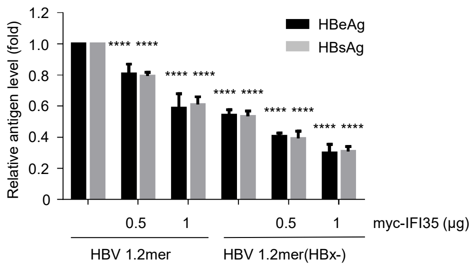

Supplementary fig 5.

(A)

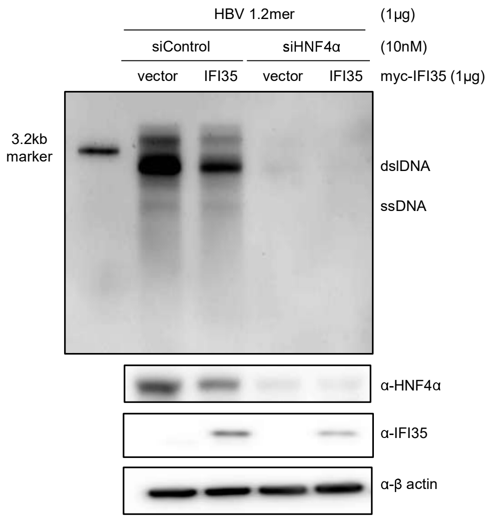

(B)

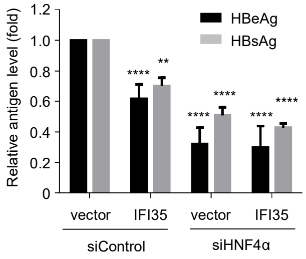

Supplementary fig 6.

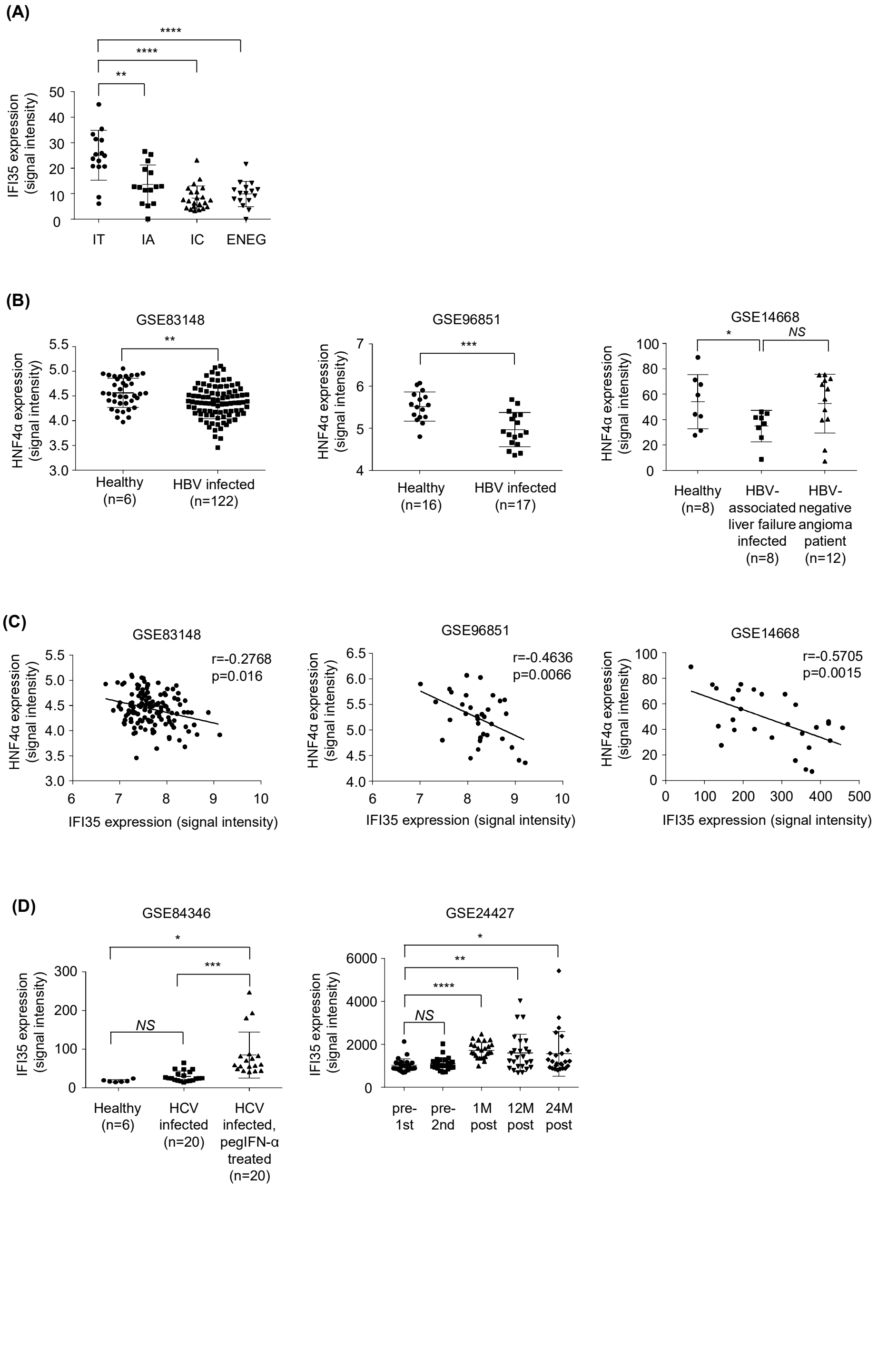

Supplement: Supplementary file 1 [file 12929_2026_1239_MOESM1_ESM.pdf]
