# Supplementary material for: IFI35 suppresses the transcription of hepatitis B virus cccDNA minichromosome via promoting HNF4α proteasomal degradation
Source: J Biomed Sci. 2026 Mar 30;33:36. doi: 10.1186/s12929-026-01239-w (PMC13034610; doi:10.1186/s12929-026-01239-w)

IFI35 suppresses the transcription of hepatitis B virus cccDNA minichromosome via promoting HNF4α proteasomal degradation

uncropped data

Fig 2.

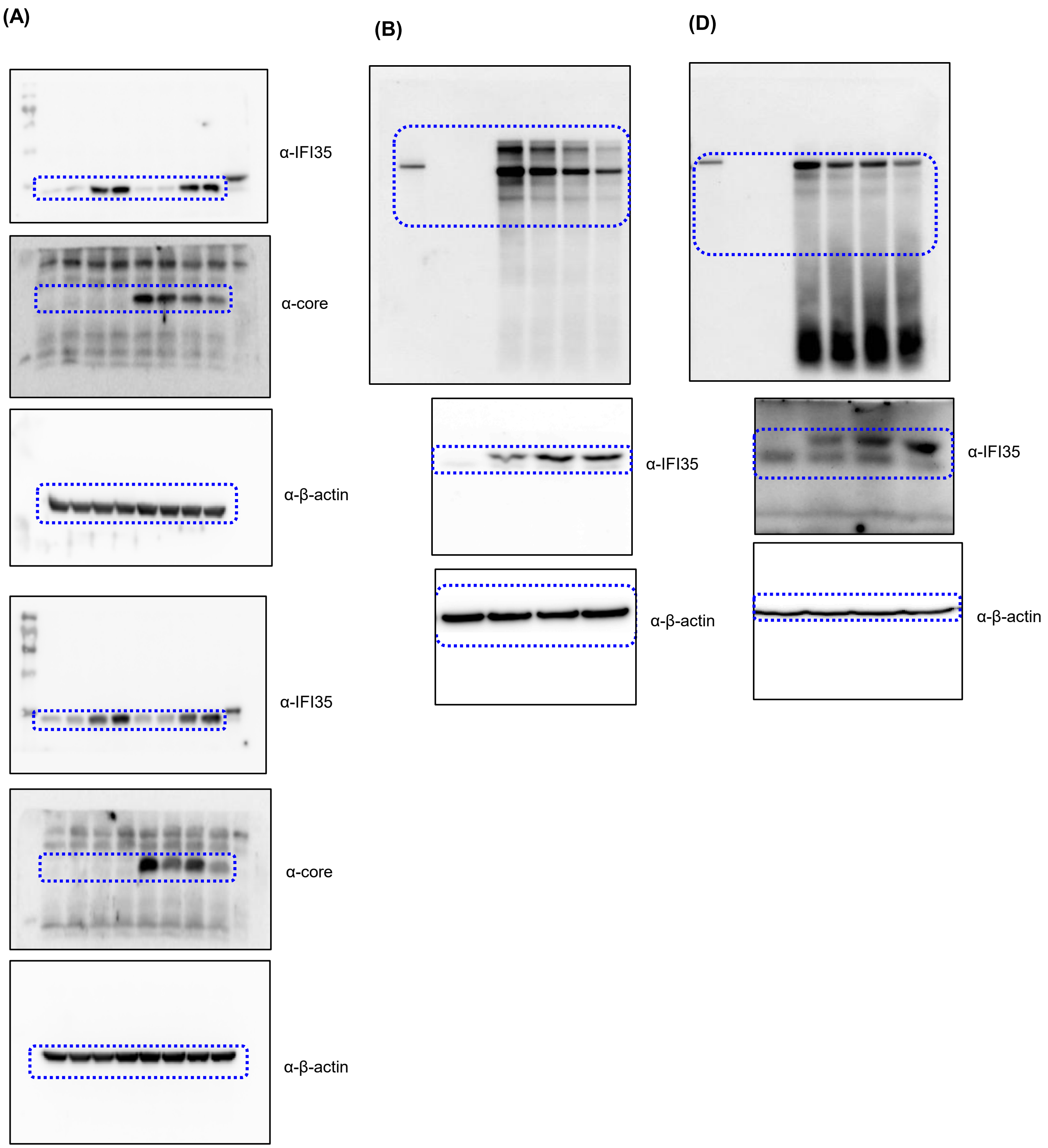
(A)
α-IFI35
α-core
α-β-actin
α-IFI35
α-core
α-β-actin
(B)
α-IFI35
α-β-actin
(D)
α-IFI35
α-β-actin

Fig 3.

(A)

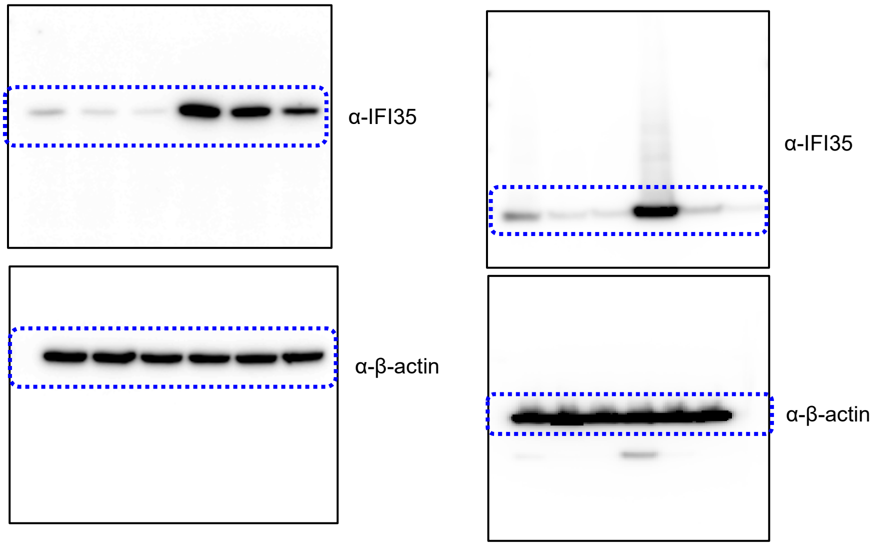

(B)

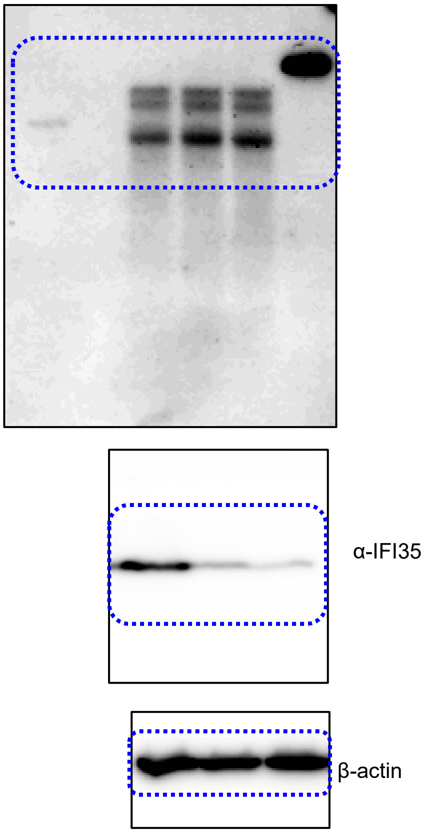

(D)

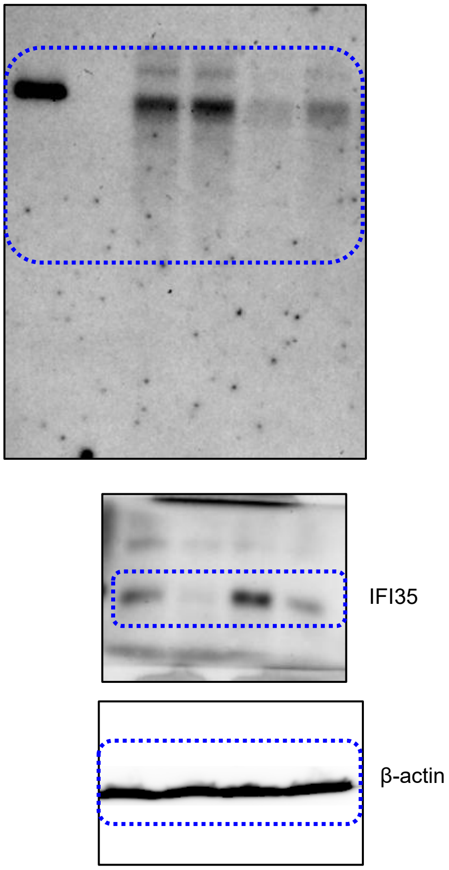

Fig 4.

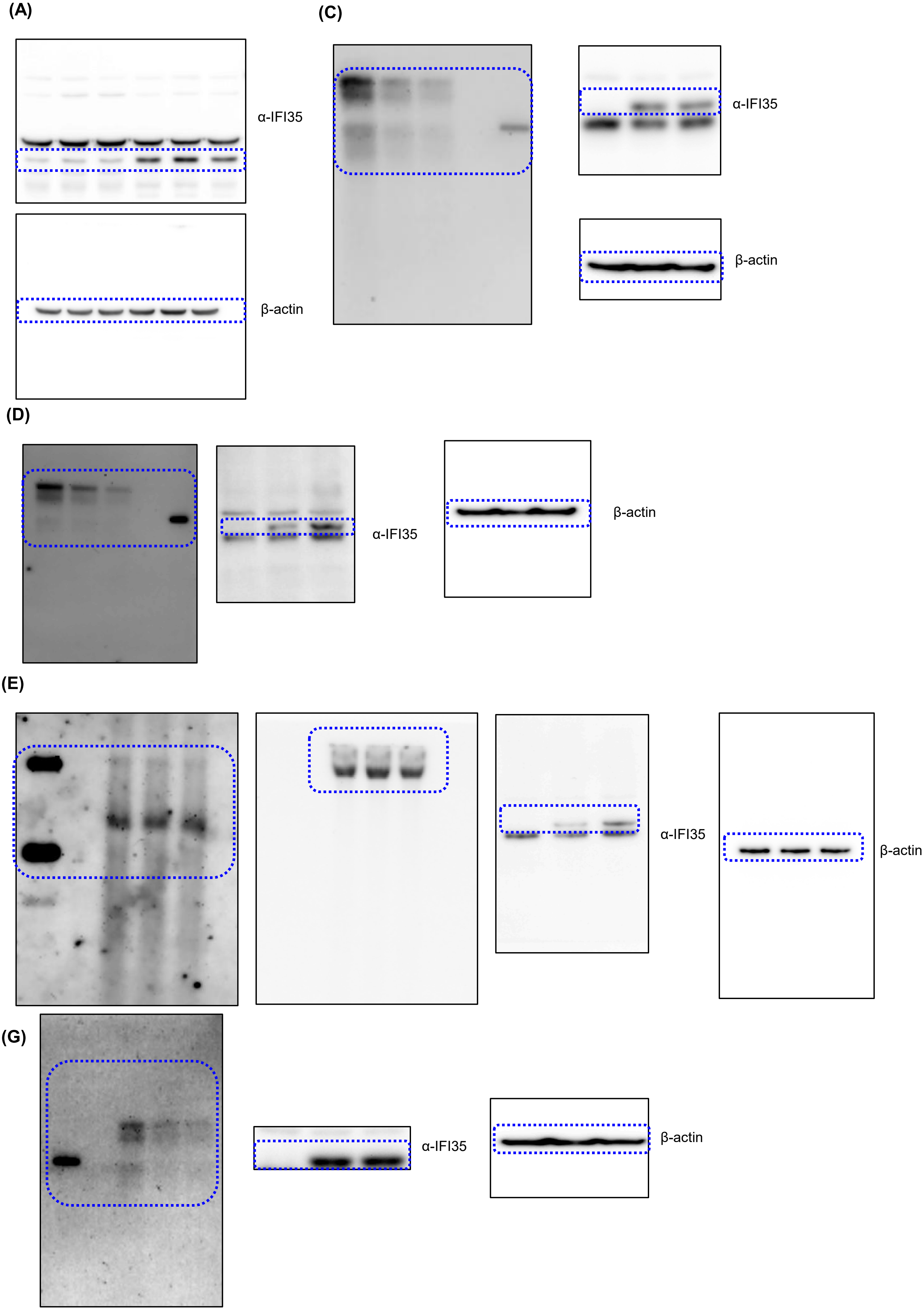
(A)
α-IFI35
β-actin
(C)
α-IFI35
β-actin
(D)
α-IFI35
β-actin
(E)
α-IFI35
β-actin
(G)
α-IFI35
β-actin

Fig 5.

(A)

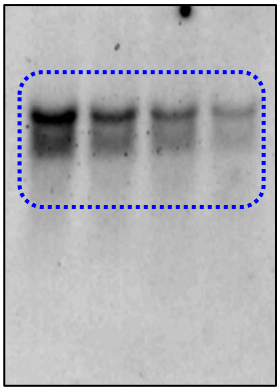

Fig 6.

(A)

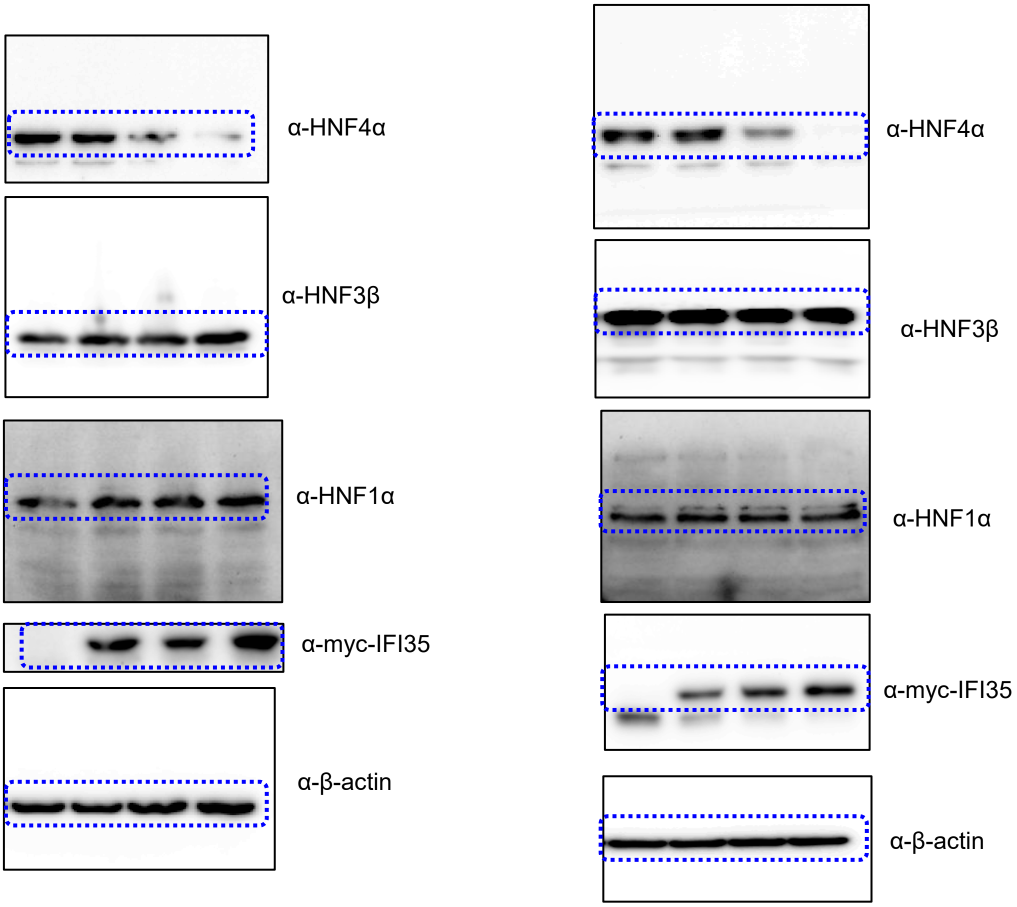
α-HNF4α
α-HNF3β
α-HNF1α
α-myc-IFI35
α-β-actin
α-HNF4α
α-HNF3β
α-HNF1α
α-myc-IFI35
α-β-actin

(C)

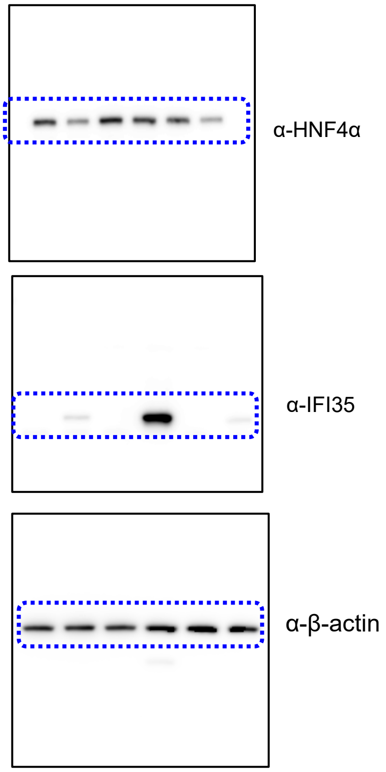
α-HNF4α
α-IFI35
α-β-actin

Fig 6.

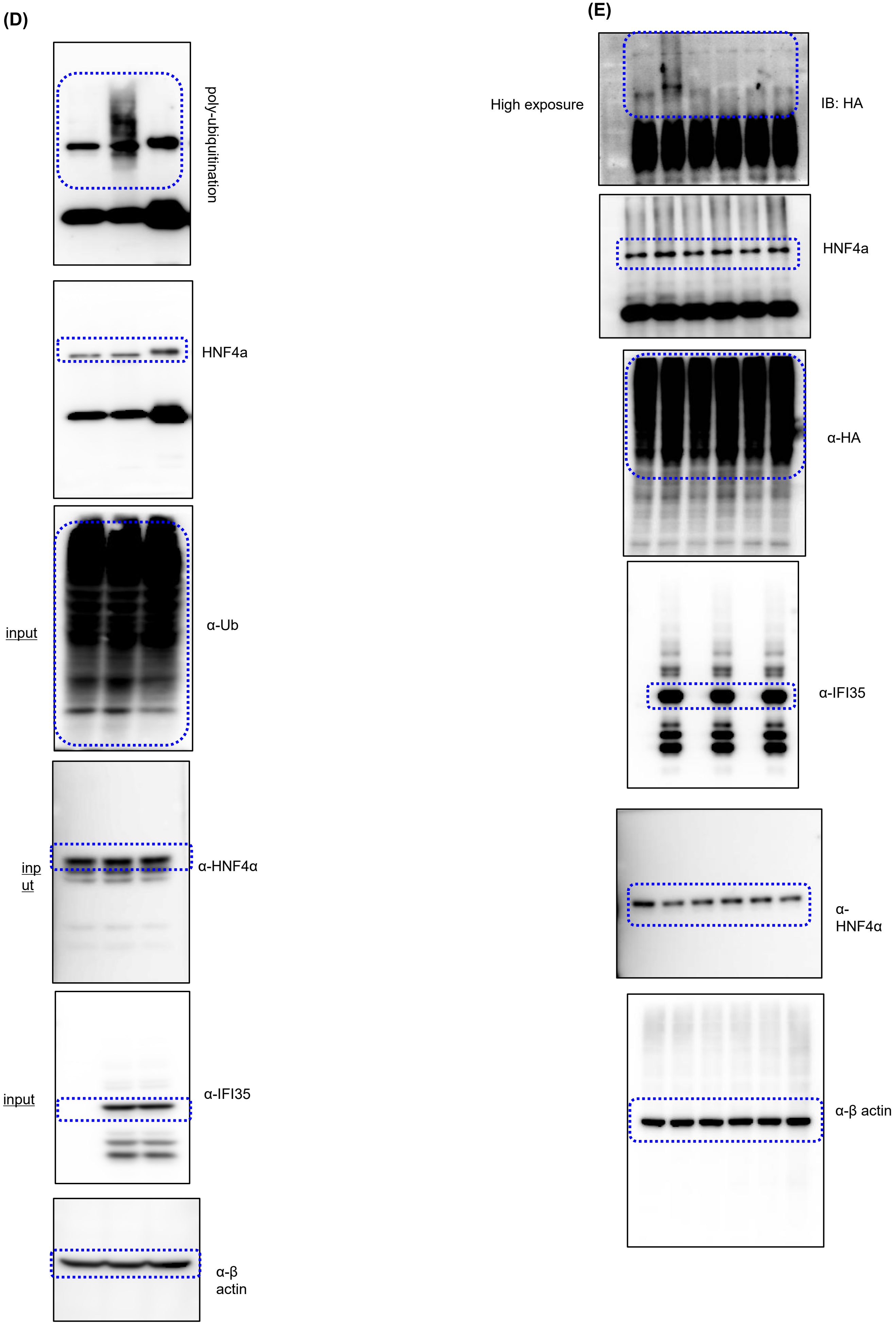
(D)
poly-ubiquitination
HNF4a
input
α-Ub
inp
ut
α-HNF4α
input
α-IFI35
α-β
actin
(E)
High exposure
IB: HA
HNF4a
α-HA
α-IFI35
α-
HNF4α
α-β actin

Fig 6.

(F)

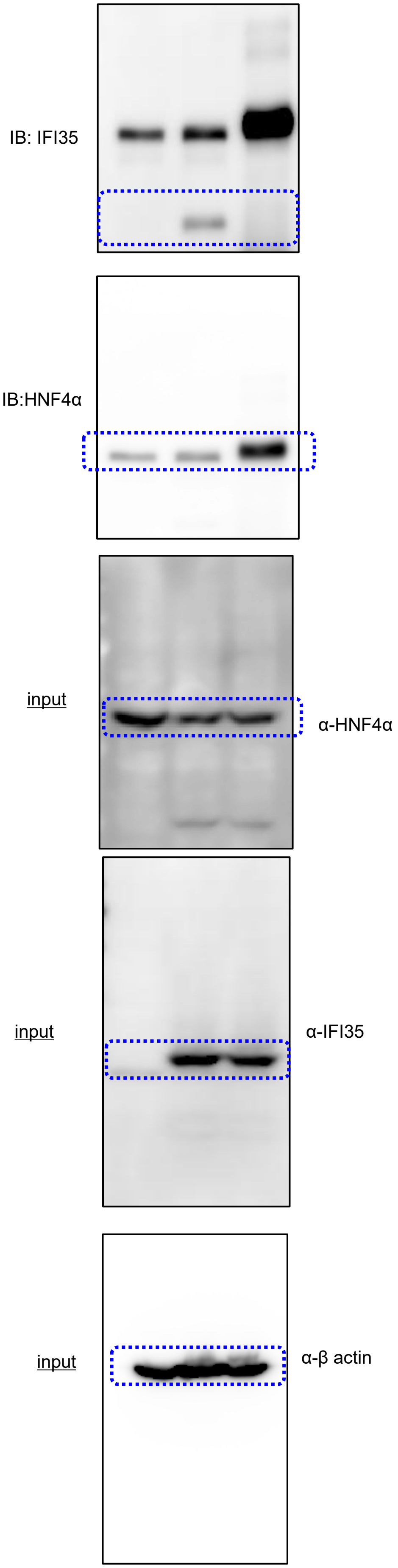
IB: IFI35
IB:HNF4α
input
α-HNF4α
input
α-IFI35
input
α-β actin

(G)

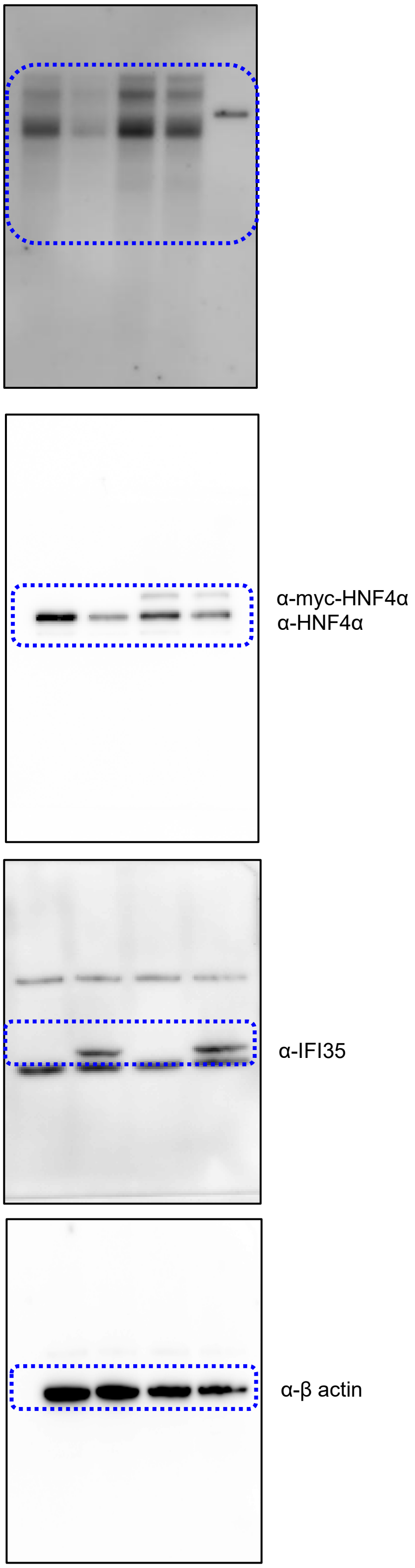
α-myc-HNF4α
α-HNF4α
α-IFI35
α-β actin

Fig 7.

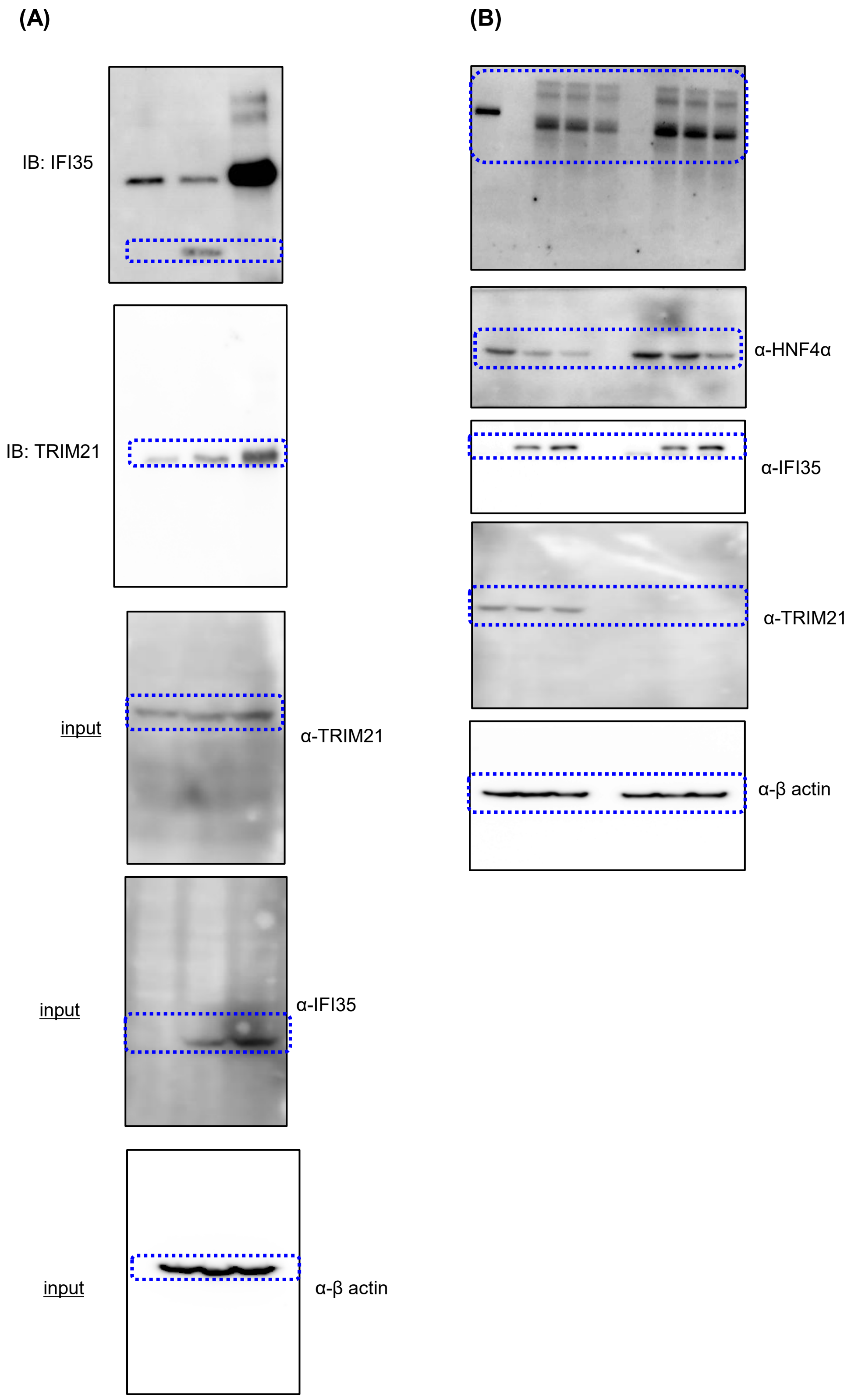
(A)
IB: IFI35
IB: TRIM21
input
α-TRIM21
input
α-IFI35
input
α-β actin
(B)
α-HNF4α
α-IFI35
α-TRIM21
α-β actin

Fig 7.

(E)

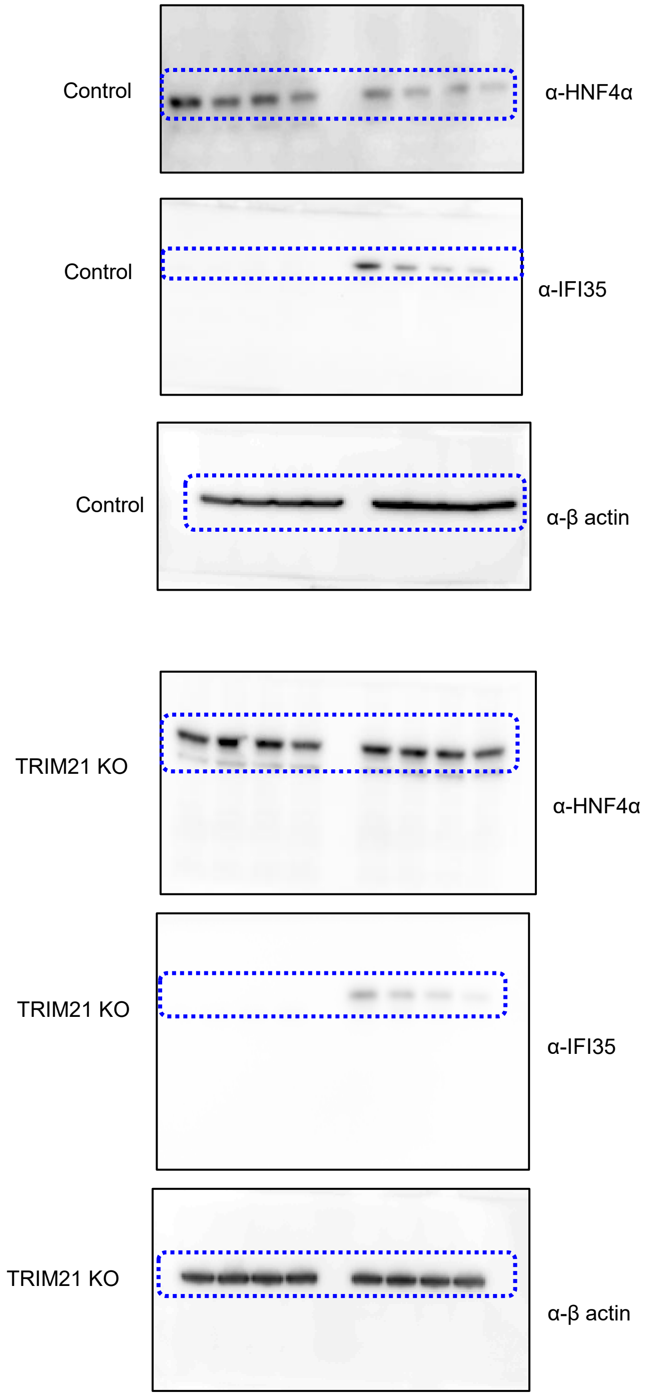

Control
α-HNF4α
Control
α-IFI35
Control
α-β actin
TRIM21 KO
α-HNF4α
TRIM21 KO
α-IFI35
TRIM21 KO
α-β actin

Fig 8.

(B)

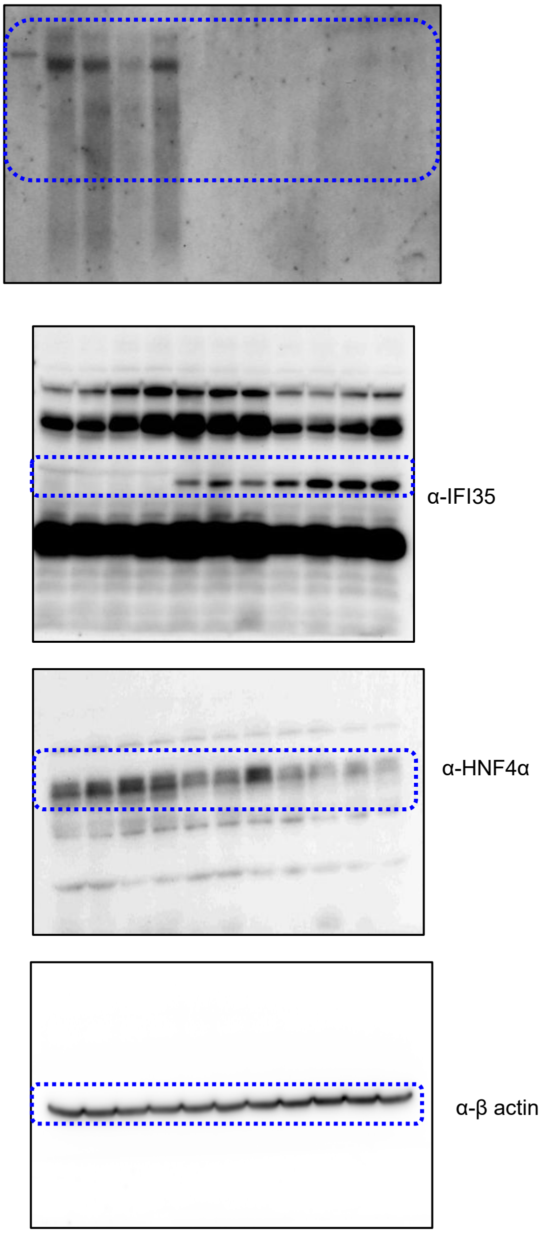

Supplementary fig 2.

(A)

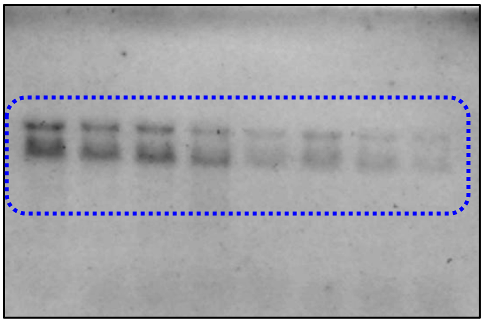

Supplementary fig 4.

(B)

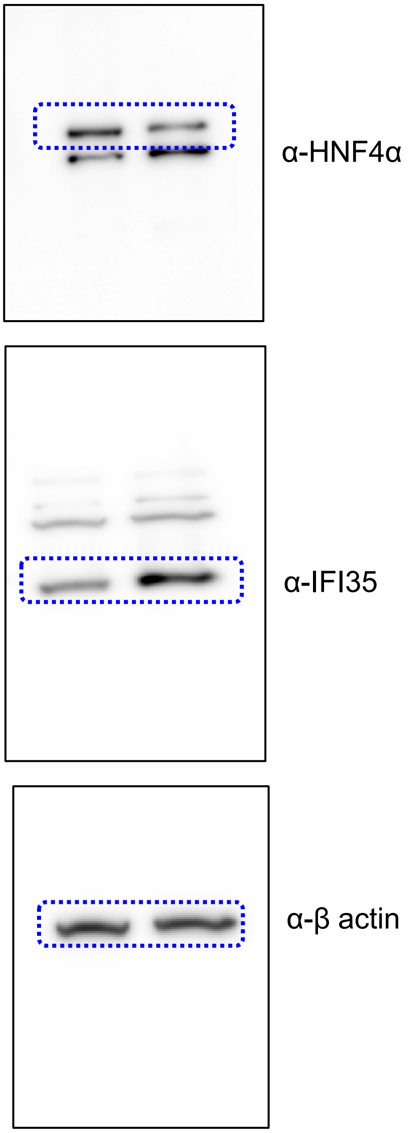

Supplementary fig 5.

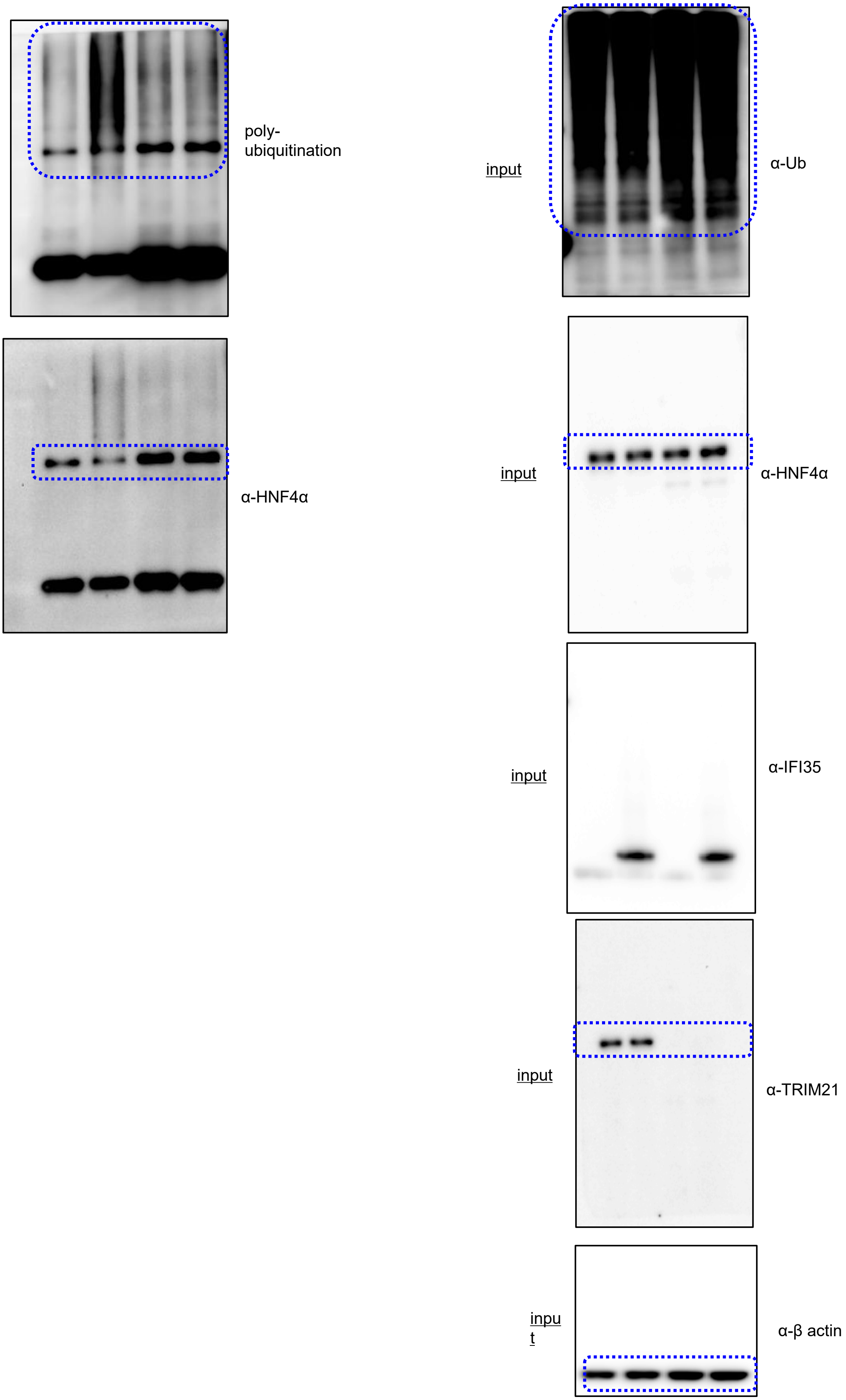

Supplementary Fig 1

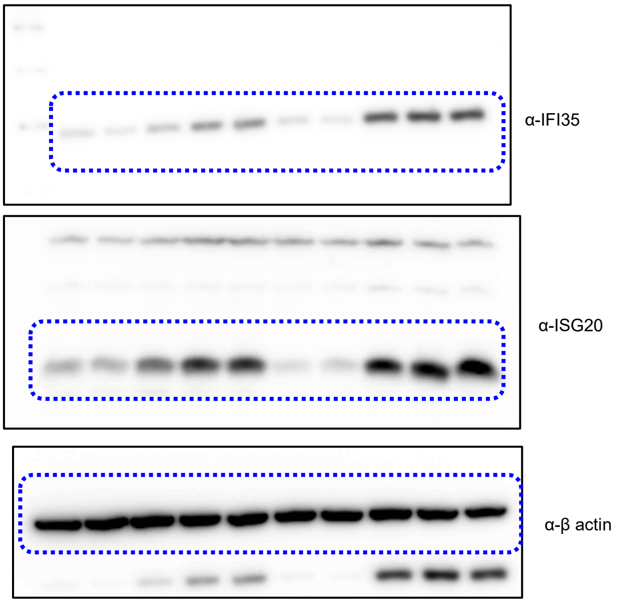

Supplementary Fig 5

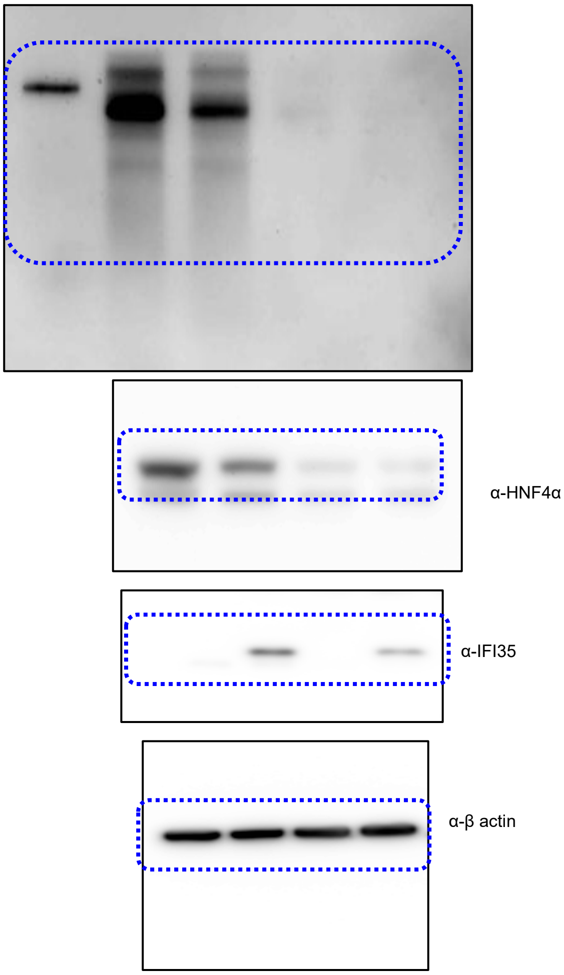

Fig 7G

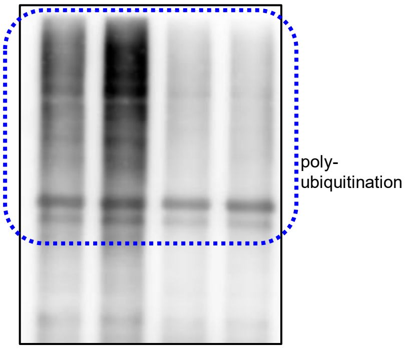

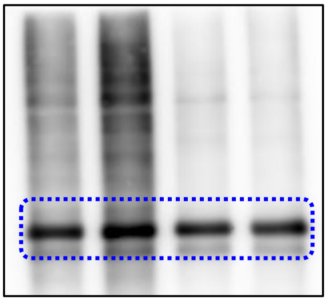

Fig 9C

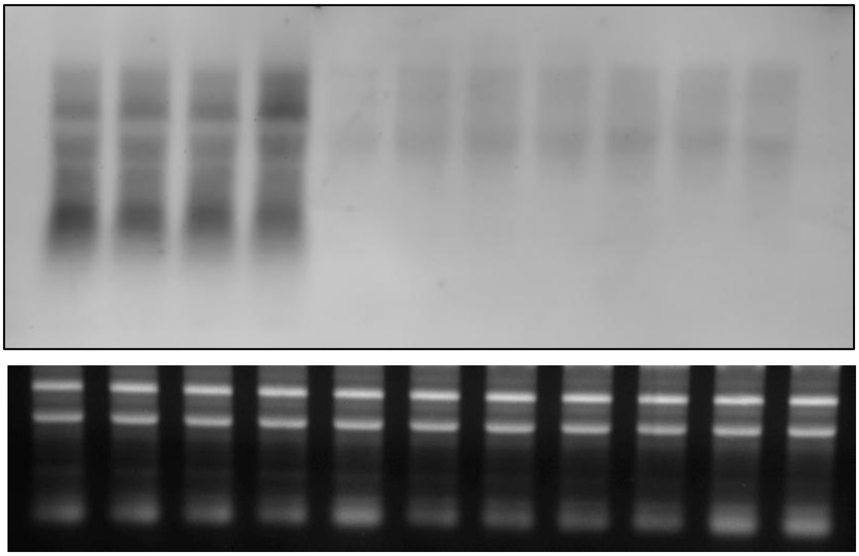

Supplementary Fig 1B

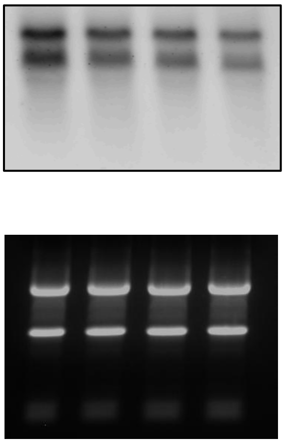

Fig 3F

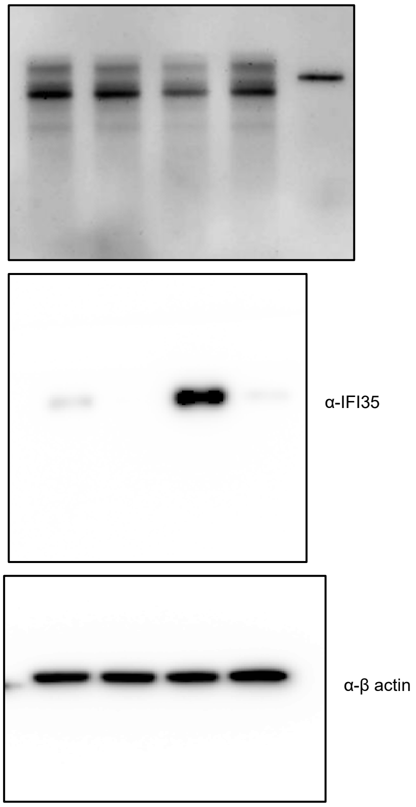

Fig. 8A

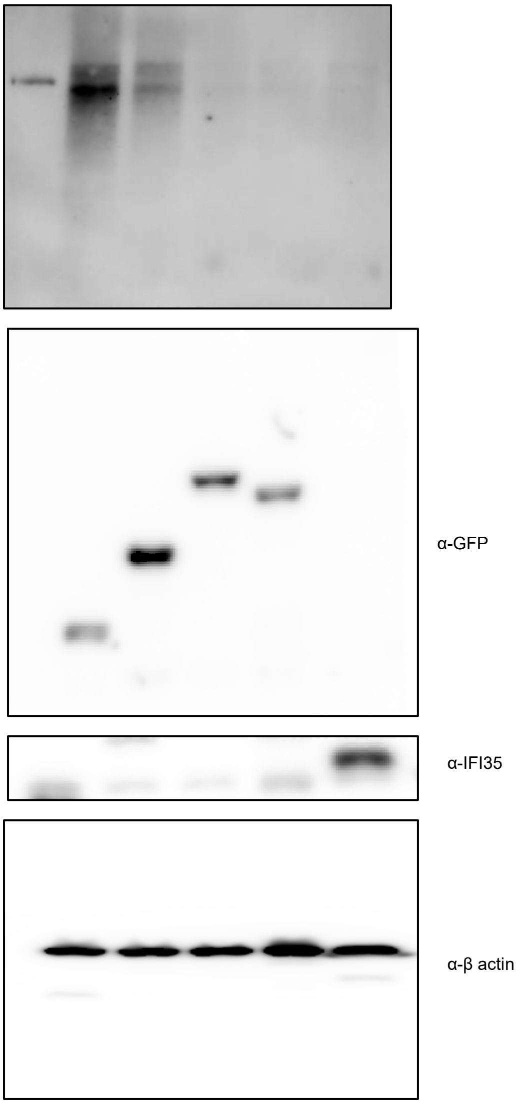

Fig 8D

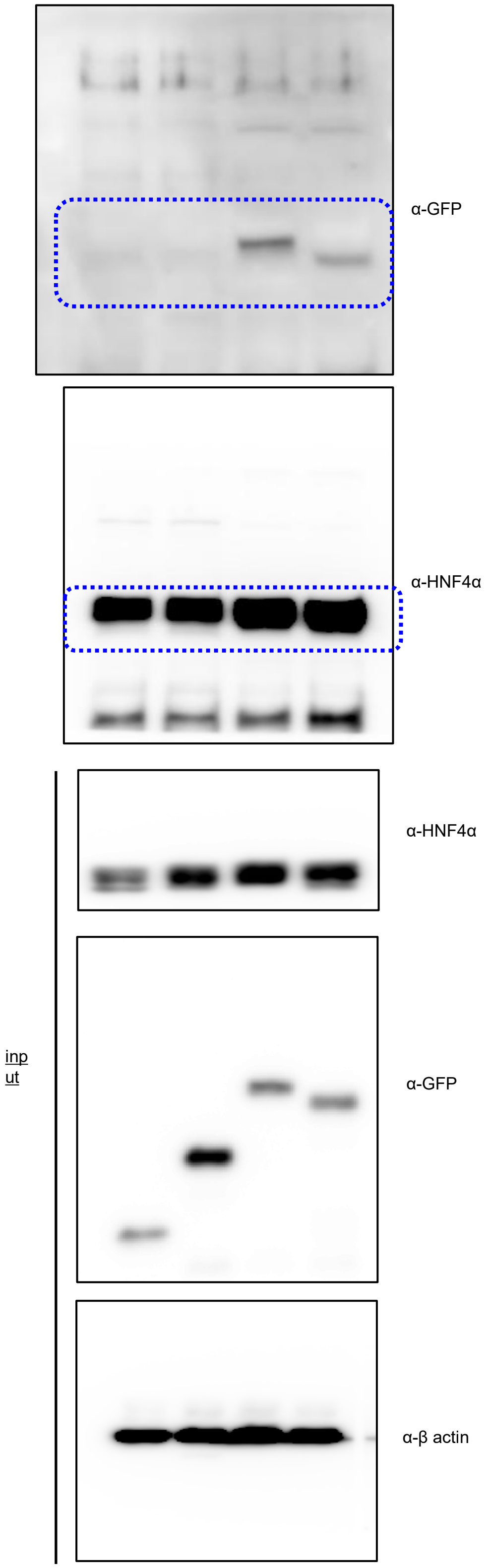
α-GFP
α-HNF4α
α-HNF4α
α-GFP
α-β actin
input

Fig 8E

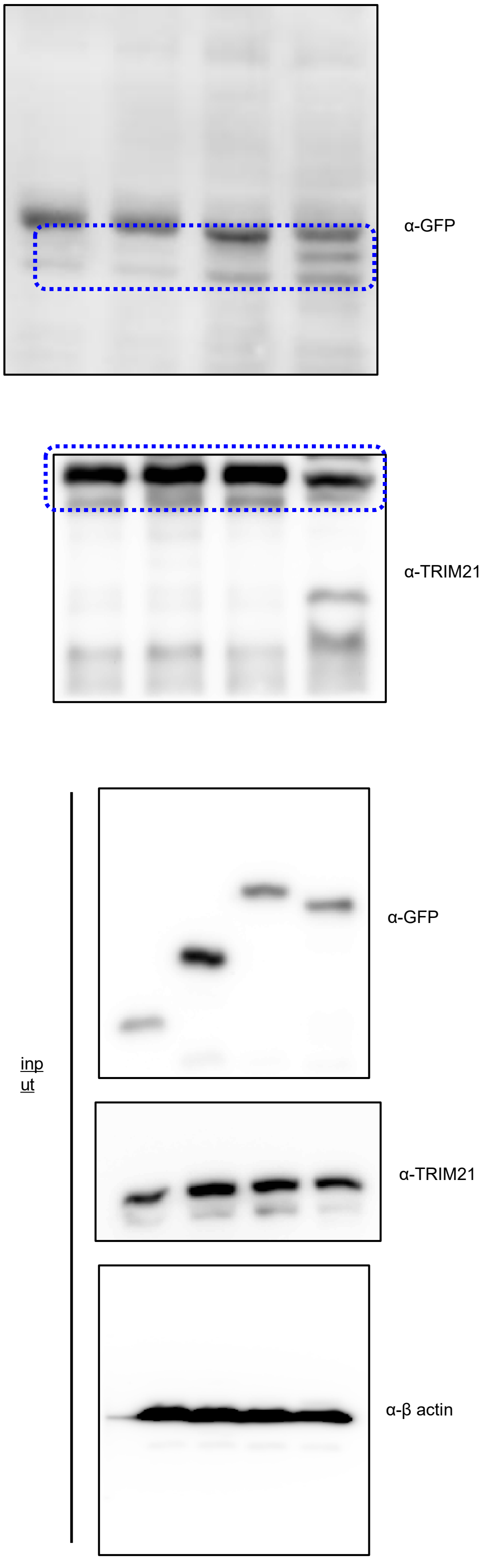
α-GFP
α-TRIM21
α-GFP
α-TRIM21
α-β actin
input

Supplementary Fig. 4

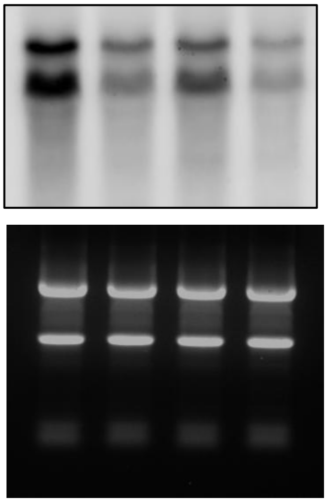

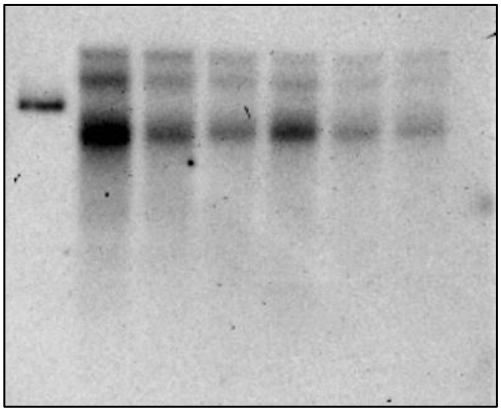

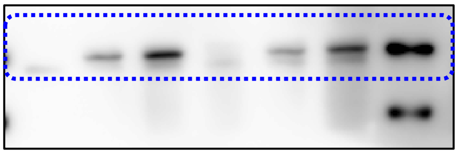

α-IFI35

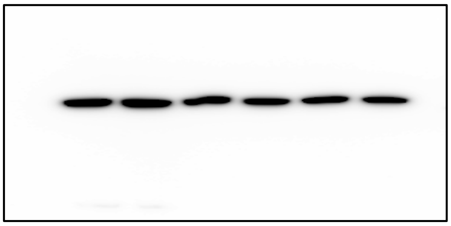

α-β actin

Fig. 6H

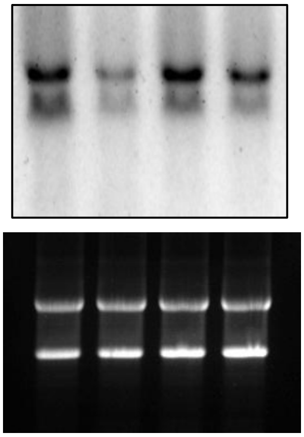

Fig. 7E

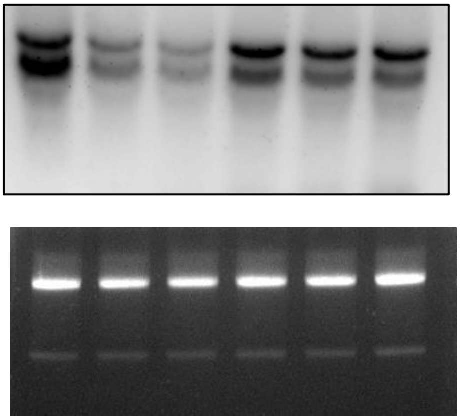

Supplement: Supplementary file 2 — Figure 1. IFN-γ strongly induces IFI35 expression and reduces HBV transcription in HepG2 cells. (A) HepG2 cells were transfected with HBV 1.2mer (1μg) and subsequently treated with concentrations of IFN-α or IFN-γ (100, 500, and 1000 U/ml). Whole-cell lysates were collected and analyzed by immunoblotting using the indicated antibodies. ISG20 was included as a representative interferon-stimulated gene to confirm activation of interferon signaling pathways. β-actin served as a loading control. Representative immunoblots from three independent experiments (n = 3) are shown. HepG2 cells were transfected with HBV 1.2mer (1 μg). 6 hours post-transfection, cells were treated with IFN-γ at the indicated concentrations (250, 500, and 1000 U/ml) for three times, and harvested at 72 h post-transfection. (B) HBV transcripts (pgRNA and preS/S RNA) were analyzed by Northern blotting; 18S and 28S rRNA are shown as loading controls. (C) Secreted HBeAg and HBsAg in culture supernatants were quantified and presented as relative levels normalized to the untreated control. Data represent mean ± SD (n = 3). Statistical significance was determined relative to the untreated control (*p < 0.05, ***p < 0.001, ****p < 0.0001). Figure 2. Effect of IFI35 on HBV RNA stability. (A) HepG2 cells were transfected with either control vector or IFI35 expression plasmid. At 72 hours post-transfection, cells were treated with Actinomycin D (10 μg/mL) and total RNA was harvested at 0, 2, 4, and 6 hours post treatment. HBV RNA levels were assessed by Northern blotting. Ethidium bromide staining of 28S and 18S rRNA served as loading controls. (B) Quantification of HBV RNA decay from (A) is shown. RNA degradation patterns were compared between control and IFI35-overexpressing cells following Actinomycin D treatment. Figure 3. Effect of IFI35 on SP1 and SP2 promoter activity. (A) Schematic representations of the preS1p-Luc and preS2p-Luc luciferase reporter constructs are shown. (B and [file 12929_2026_1239_MOESM2_ESM.pdf]
